# Plasma-Assisted Surface Nitridation of Proton Intercalatable WO_3_ for Efficient Electrocatalytic Ammonia Synthesis

**DOI:** 10.1021/acsenergylett.5c01034

**Published:** 2025-06-22

**Authors:** Zhiyuan Zhang, Christopher Kondratowicz, Jacob Smith, Pavel Kucheryavy, Junjie Ouyang, Yijie Xu, Elizabeth Desmet, Sophia Kurdziel, Eddie Tang, Micheal Adeleke, Aditya Dilip Lele, John Mark Martirez, Miaofang Chi, Yiguang Ju, Huixin He

**Affiliations:** † Department of Chemistry, Rutgers, the State University of New Jersey, Newark, New Jersey 07102, United States; ‡ Department of Mechanical and Aerospace Engineering, 6740 Princeton University, Princeton, New Jersey 08544, United States; § Center for Nanophase Materials Sciences, Oak Ridge National Laboratory, Oak Ridge, Tennessee 37831, United States; ∥ Applied Materials and Sustainability Sciences, 17217 Princeton Plasma Physics Laboratory, Princeton, New Jersey 08543, United States; ⊥ Montgomery High School, Skillman, New Jersey 08558, United States; # Science Park High School, Newark, New Jersey 07103, United States; ¶ Department of Mechanical Engineering, 3536 Rowan University, Glassboro, New Jersey 08028, United States; ■ Thomas Lord Department of Mechanical Engineering & Materials Science, 3065 Duke University, Durham, North Carolina 27708, United States

## Abstract

Electrocatalytic nitrogen reduction (eNRR) offers a green pathway for the production of NH_3_ from N_2_ and H_2_O under ambient conditions. Transition metal oxynitrides (TMO_*x*_N_*y*_) are among the most promising catalysts but face challenges in achieving a high yield and faradaic efficiency (FE). This work develops a hybrid WO_*x*_N_*y*_/WO_3_ catalyst with a unique heterogeneous interfacial complexion (HIC) structure. This design enables *in situ* generation and delivery of highly active hydrogen atoms (H*) in acidic electrolytes, promoting nitrogen hydrogenation and the formation of nitrogen vacancies (Nv) on the WO_*x*_N_*y*_ surface. This significantly enhances the selectivity of eNRR for NH_3_ synthesis while suppressing the hydrogen evolution reaction (HER). A simple two-step fabrication process—microwave hydrothermal growth followed by plasma-assisted surface nitridation—was developed to fabricate the designed catalyst electrode, achieving an NH_3_ yield of 3.2 × 10^–10^ mol·cm^–2^·s^–1^ with 40.1% FE, outperforming most TMN/TMO_*x*_N_*y*_ electrocatalysts. Multiple control experiments confirm that the eNRR follows an HIC-enhanced Mars–van Krevelen (MvK) mechanism.

## Graphical abstract

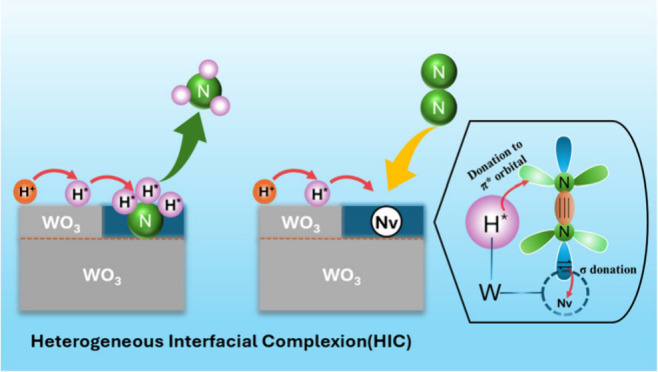

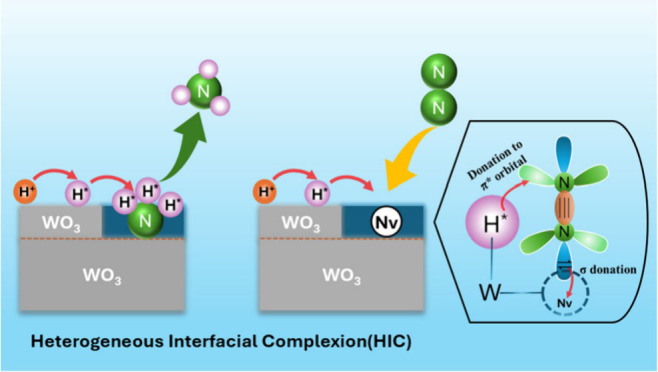

Ammonia (NH_3_) has traditionally been used as a fertilizer and a key chemical in the chemical industries. More recently, NH_3_ has emerged as a promising hydrogen (H_2_) carrier for green power storage and generation, due to its higher energy density, lower storage and transport costs, and lower flammability compared to H_2_.^1,2^ However, the current production method, the Haber–Bosch (H–B) process, operates under high temperatures (∼500 °C) and pressures (∼250 atm) and relies on H_2_ produced from fossil fuels. This makes it not only energy- and CO_2_-intensive but also highly centralized, limiting its integration with intermittent and location-specific renewable electricity.^3,4^ To address these challenges, it is essential to develop efficient, distributed, and electrified methods to produce green NH_3_ directly from water (H_2_O) by using renewable electricity.^5,6^

To overcome the challenges of the H–B process and enable electrified synthesis of NH_3_, plasma-catalytic NH_3_ synthesis from N_2_ and water (H_2_O) or H_2_ has been pursued.^7–9^ Plasma catalysis offers nonequilibrium reaction pathways for NH_3_ synthesis by utilizing active atomic nitrogen (N) and vibrationally excited nitrogen (N_2_(ν)), as well as catalyst surface nitridation.^7,8,10^ However, the energy efficiency of plasma-assisted NH_3_ synthesis remains significantly lower (8.7 g-NH_3_/kWh) than that of the H–B process (500 g-NH_3_/kWh).^11,12^ Additionally, when H_2_O is used as the hydrogen source in plasma, undesired byproducts such as NO_*x*_ and H_2_O_2_ can be generated.^10^ Electrocatalytic nitrogen reduction reaction (eNRR) allows green production of NH_3_ from N_2_ and H_2_O under ambient conditions without the undesired pollutants and other harmful byproducts.^13^ However, the practical implementation of eNRR for NH_3_ production is impeded by the extremely low NH_3_ yield and low Faradaic efficiency (FE) due to the high activation energy of inert N_2_ molecules and the overwhelming HER competition reaction.^14,15^

Transition metal nitrides (TMNs) and oxynitrides (TMO_*x*_N_*y*_) have emerged as promising catalysts for eNRR due to their distinctive structural properties, which enable nitrogen reduction through the Mars–van Krevelen (MvK) catalytic pathway with much lower energy barrier compared to N adsorbate reduction via direct proton-coupled electron transfer (PCET).^16–20^ In the MvK pathway, ammonia (NH_3_) is initially produced by hydrogenating lattice nitrogen (N) atoms on the surface of TMNs or TMO_*x*_N_*y*_. This process significantly reduces the energy barrier for NH_3_ production as it avoids the need for direct activation and cleavage of the N≡N triple bond in N_2_.^14^ Desorption of these NH_3_ molecules generates nitrogen vacancies (Nv) on the catalyst surface, which subsequently act as the catalytic sites, adsorbing and activating the dissolved N_2_ from the electrolyte and completing the catalytic cycles. Although the subsequent steps still require the cleavage of the N≡N triple bond, it is noteworthy that the nitrogen vacancies not only provide unsaturated coordination sites that facilitate N_2_ adsorption but also efficiently activate the adsorbed N_2_ molecules by accommodating the lone pair of electrons from N_2_ due to the electron-deficient nature of the vacancies.^21^ Importantly, the electron-deficient nature of nitrogen vacancies selectively favors the adsorption and activation of N_2_ over H^+^, thereby minimizing competition from hydrogen evolution reaction (HER).^16,17^ The concurrent high activity and selectivity for eNRR stands in stark contrast to oxygen vacancies (Ov) on transition metal oxides (TMO).^22^

Recent studies demonstrated that transition metal oxynitrides (TMO_*x*_N_*y*_) exhibit higher catalytic activity and better stability than that of TMN-based catalysts.^2,17,18,22,23^ Nevertheless, the NH_3_ yield for both TMO_*x*_N_*y*_- and TMN-based catalysts is still far lower for practical applications. Considerable efforts have been made to understand the reasons behind the observed low eNRR activities.^22,24^ It is commonly accepted that Nv generation/regeneration is considered as the rate-determining step of the MvK mechanism.^17,22,25^ For Nv-free TMN and TMO_*x*_N_*y*_ catalysts, the initial hydrogenation of lattice nitrogen is widely regarded as the most challenging step.^22,24–27^ For Nv-rich TMN and TMO_*x*_N_*y*_ catalysts, the desorption of the generated NH_3_ becomes a critical factor in completing the Nv regeneration cycle.^21,24^

To promote Nv generation, the Hosono group developed nickel-loaded lanthanum nitride (LaN) for high-temperature thermal catalytic hydrogenation of N_2_ for NH_3_ synthesis.^27^ In this catalyst design, nickel clusters efficiently dissociate H_2_ into highly active H* at high temperatures, which then migrate to the LaN, significantly facilitating nitrogen atom hydrogenation and promoting the formation of nitrogen vacancies (Nv) on the LaN surface. In the electrocatalytic field, Zhang’s group has demonstrated the crucial role of H* in facilitating Nv generation on copper nitride (Cu_3_N) at room temperatures.^28^ Their catalyst design employed a novel graphdiyne support to electrochemically generate H atoms. The coexistence of H atoms and Nv significantly facilitated the electrocatalytic reduction of nitrate (NO_3_^^–^^). However, leveraging H* to induce Nv formation in TMN/TMO_*x*_N_*y*_ systems for enhanced reduction of electrocatalytic N_2_ to NH_3_ remains unexplored.

In this work, we design WO_*x*_N_*y*_/WO_3_ hybrid electrochemical catalysts featuring a unique heterogeneous interfacial complexion (HIC) structure^29^ (Scheme 1a). We hypothesize that the HIC structure enables the *in situ* generation of H* via proton intercalation, enhancing eNRR through an HIC-enhanced Mars–van Krevelen (MvK) mechanism. WO_*x*_N_*y*_ is selected for its eNRR activity and stability against chemical decomposition, outperforming tungsten nitride (WN) due to its elevated N_2p_ orbital near the Fermi level (E_F_).^2,22,30^ Meanwhile, WO_3_ facilitates *in situ* proton (H^+^) intercalation (WO_3_+ *x*H^+^ + *x*e^–^ ⇌ H_*x*_WO_3_), generating mobile, active, and relatively long-lived H atoms (in the form of weakly bound hydrogen, denoted as W–H*) in acidic electrolyte.^31–33^ This HIC architecture ensures that WO_3_ retains its proton intercalation capability along the electron–proton transport pathway. We further hypothesize that W–H* generated at the WO_*x*_N_*y*_/WO_3_ interface or migrated from WO_3_ domains to this interface promotes hydrogenation of lattice nitrogen in WO_*x*_N_*y*_ to form NH_3_. The abundance of H^+^ ions in the acidic electrolyte facilitates the protonation and desorption of the produced NH_3_ from the WO_*x*_N_*y*_ catalyst surface, facilitating the generation of nitrogen vacancies (Nv). In the subsequent steps, these Nv sites, combined with the continuous H* supply at neighboring W centers, form new catalytic sites, as illustrated in Scheme 1b. The newly formed catalytic sites at the WO_*x*_N_*y*_/WO_3_ interface facilitate bidirectional electron transfer, a critical requirement for efficient N_2_ activation, thereby significantly enhancing activation capability.^34^ Specifically, the Nv preferentially adsorbs an electron donor like N_2_ over H^+^ due to their electron-deficient nature,^21^ allowing Nv to accept the lone pair electrons from adsorbed N_2_. Meanwhile, the H* on adjacent W sites donates electrons into the antibonding π orbitals (π*) of the adsorbed N_2_, weakening the N≡N triple bond and significantly enhancing its activation.^35,36^ This cooperative interaction boosts N_2_ activation, increasing the level of selective ammonia synthesis while suppressing the competing hydrogen evolution reaction (HER).

**Scheme 1:**
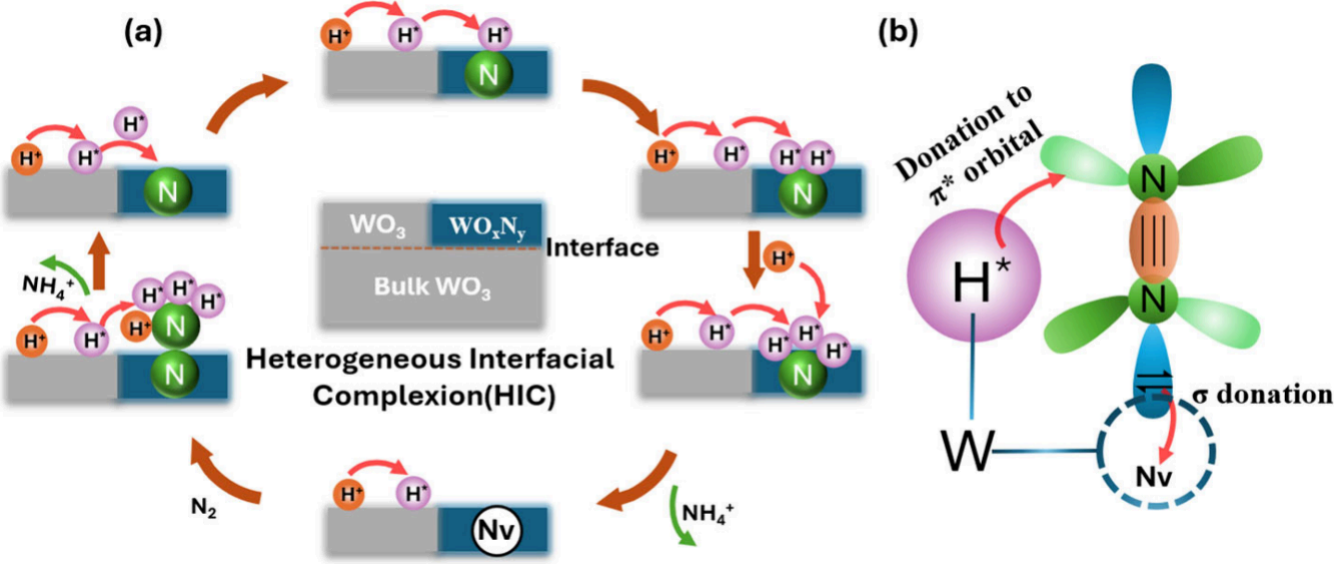
(a) Schematic illustration showing how the designed WO_*x*_N_*y*_/WO_3_ electrocatalyst with an HIC structure enables *in situ* generation of H* via proton intercalation to promote the hydrogenation of lattice nitrogen (N) in WO_*x*_N_*y*_, leading to the formation of nitrogen vacancies (Nv) and the creation of new catalytic centers for enhanced eNRR. (b) Schematic representation of the orbital interaction between N_2_ and the new catalytic center, which is highly active and selective for N_2_ absorption and activation.

To fabricate the WO_*x*_N_*y*_/WO_3_ hybrid catalysts with the proposed HIC architecture, a novel two-step process was developed. This process involves the facile microwave-assisted hydrothermal growth of a WO_3_ nanosheet array, followed by a surface nitridation process achieved through nonequilibrium hydrogen/nitrogen plasma-assisted treatment. As illustrated in Scheme 2(a,b), a predominantly vertically aligned WO_3_ nanosheet array is first grown on a highly conductive carbon cloth with a hydrophobic microporous layer (MPL) using a facile microwave-assisted hydrothermal method. This is followed by a plasma-assisted surface nitridation process to convert part of the outer layer of WO_3_ into WO_*x*_N_*y*_ while preserving the bulk WO_3_ structure. Upon optimization, the developed WO_*x*_N_*y*_/WO_3_ catalyst electrode achieved an impressive NH_3_ yield rate of 3.2 × 10^–10^ mol·cm^–2^·s^–1^ at −0.15 V vs RHE in an acidic H_2_SO_4_ electrolyte (pH = 2). This yield is approximately eight times higher than that of a single-layer 2D W_2_N_3_ catalyst, which theoretically possesses significantly more catalytic centers due to its single-layer structure.^21^ Remarkably, this high yield is accompanied by an unprecedented Faradaic efficiency (FE) of 40.1%, the highest reported for any transition metal nitride (TMN) or transition metal oxynitride (TMO_*x*_N_*y*_)-based catalyst, which typically exhibit FE values below 15%.^17,21,22,37–41^

**Scheme 2:**
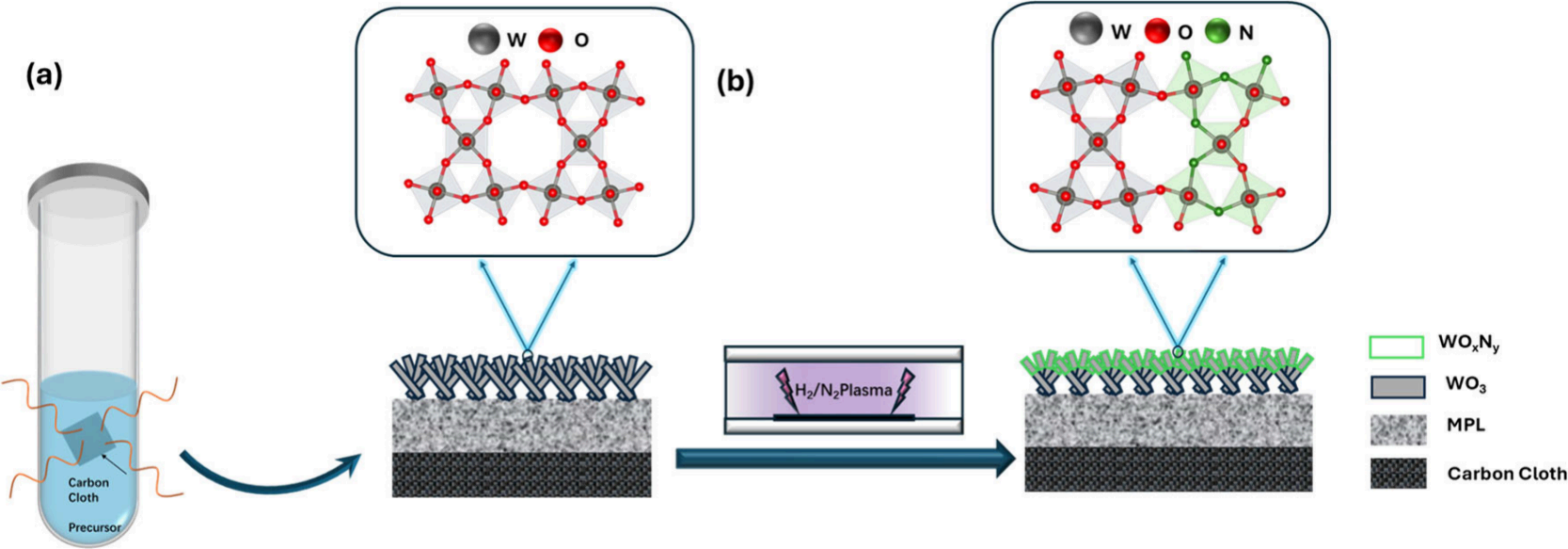
Schematic illustration of the two-step process for preparing largely vertically aligned WO_*x*_N_*y*_/WO_3_ with the designed HIC architecture on a piece of carbon cloth with a hydrophobic MPL. (a) Microwave-assisted hydrothermal growth of WO_3_ nanosheet array on the MPL of carbon cloth; (b) surface nitridation of the WO_3_ nanosheets via nonequilibrium plasma to form WO_*x*_N_*y*_/WO_3_ while leaving the WO_3_ crystal structure and the underneath hydrophobic MPL intact. Therefore, the as-prepared WO_*x*_N_*y*_/WO_3_ hybrid catalyst electrodes were directly employed as gas diffusion electrodes for eNRR.

The detailed procedures for the growth of WO_3_ nanosheet array on a piece of highly conductive carbon cloth with a microporous layer (MPL) were described in supplementary S1. This approach significantly shortens the reaction time to just 15 min, a marked improvement compared to the 24–48 h required by conventional hydrothermal methods.^42^ Notably, this synthesis method enables us to directly grow WO_3_ on the hydrophobic MPL side of the carbon cloth.

Scanning electron microscopy (SEM) was used to characterize the structure of the WO_3_ nanostructures. Figures 1a shows the nanosheet-like morphology of the as-prepared WO_3_, with the nanosheets predominantly aligned vertically but exhibiting random orientations. Each individual nanostructure appears as a thin, platelet-like crystal measuring a few tens to hundreds of nanometers in thickness and extending laterally on the order of hundreds of nanometers to a few micrometers. Figure 1b further reveals that each nanosheet is composed of an assembly of nanocrystals, suggesting a large surface area, which is highly beneficial for catalytic applications. The crystal structure of WO_3_ was characterized with powder X-ray diffraction (PXRD). As shown in Figure 1c, the diffraction peaks of the as-prepared WO_3_ align well with those of hexagonal WO_3_ (h-WO_3_, JCPDS No. 33–1387).

**Figure 1:**
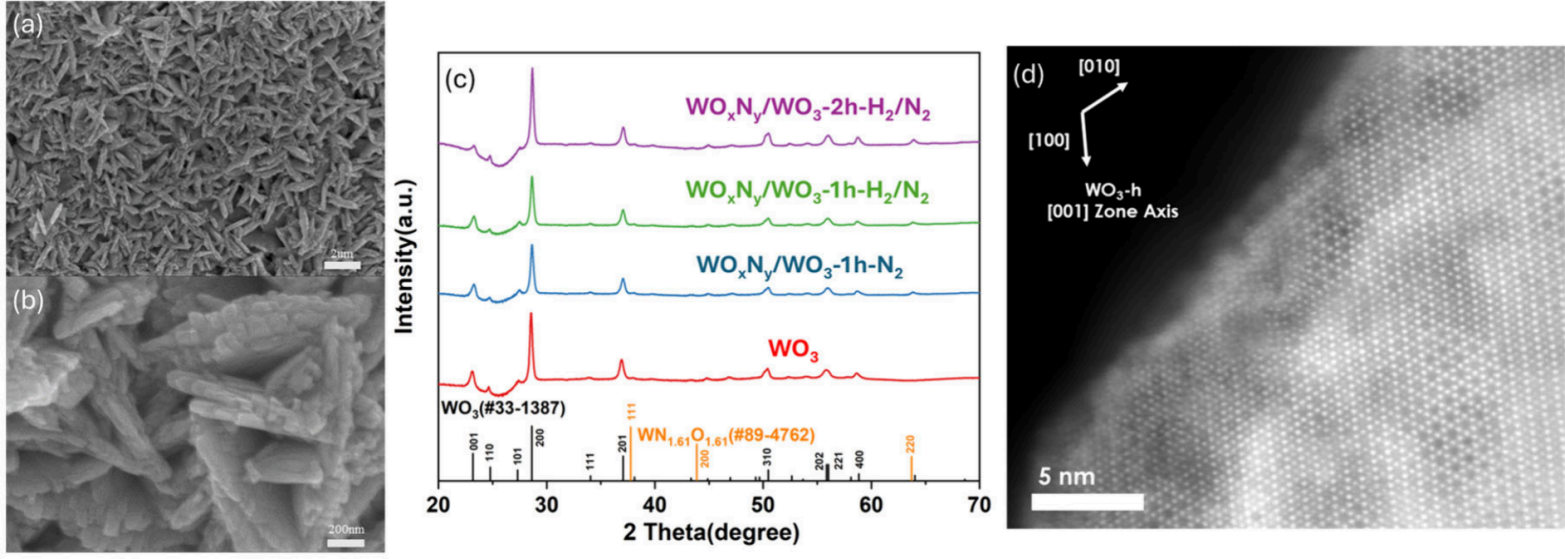
(a,b) SEM images of the h-WO_3_ nanosheets at different magnifications, which directly grow on a piece of carbon cloth with a hydrophobic microporous layer. (c) PXRD patterns of h-WO_3_ before and after plasma-assisted nitridation. (d) Surface reconstruction of h-WO_3_ after plasma treatment. Atomic-resolution HAADF-STEM imaging shows that the hexagonal structure of h-WO_3_ remains intact, with a thin amorphous layer formed on the surface.

To achieve surface nitridation of the h-WO_3_ nanosheet array while preserving the underlying WO_3_ crystal structure and the MPL layer on the carbon cloth, a novel room-temperature, nonequilibrium plasma-assisted surface nitridation approach was developed. This method utilizes a hydrogen/nitrogen mixture plasma, enabling the direct fabrication of the hybrid WO_*x*_N_*y*_/WO_3_ catalyst electrode with a designed HIC architecture at room temperature. This approach ensures precise control over nitridation while maintaining the structural integrity and electronic properties essential for efficient proton and electron transport. Briefly, the synthesized WO_3_ nanosheets on the carbon cloth described above were used as the precursor, directly located in a H_2_/N_2_ dielectric barrier discharge (DBD) plasma chamber for the surface nitridation. Various plasma treatment conditions, including N_2_ plasma for 1 h, first H_2_ plasma for a half hour, followed by N_2_ plasma for 1 h, H_2_/N_2_ (1:4) plasma for 1 h, and H_2_/N_2_ plasma for 2 h, were tested to optimize the surface nitridation. The resulting hybrid WO_*x*_N_*y*_/WO_3_ catalyst electrodes were accordingly named WO_3_-N_2_-1h, WO_3_-H_2_-0.5h–N_2_-1h, WO_3_-H_2_/N_2_-1h, and WO_3_-H_2_/N_2_-2h, respectively.

SEM and PXRD were also used to study their morphology and crystal structural change after plasma treatment. By comparing Figure S1a-c (SEM as-prepared WO_3_) to Figure S1d-f (SEM postnitridation), the WO_3_ nanostructures retain their morphology without significant changes under the applied plasma nitridation conditions. Note that a new peak at 63.6° with very low intensity appears on the PXRD spectra **(** Figure 1c) with WO_3_-H_2_/N_2_-2h having the highest intensity, indicating the formation of WO_*x*_N_*y*_ (JCPDS No. 89–4762). However, the diffraction peaks of h-WO_3_ remained largely unchanged across all of the plasma nitridation conditions studied. This result suggests that the crystal structure of h-WO_3_ remains largely intact. To further investigate the atomic-scale structural changes of h-WO_3_ after nitridation, high-angle annular dark-field scanning transmission electron microscopy (HAADF-STEM) was utilized to image the WO_3_-H_2_/N_2_-2h catalyst, which exhibited the highest intensity of the new peak at 63.6° in the PXRD spectrum (Figure 1c). As shown in Figure 1d, the well-defined hexagonal crystal structure of h-WO_3_ was largely preserved, consistent with bulk PXRD studies. However, a very thin (1–2 nm) amorphous surface layer appeared, which was likely associated with the formation of WO_*x*_N_*y*_ species, as confirmed by electron energy-loss spectroscopy (EELS). As shown in Figure S2, minimal nitrogen signals are observed in the bulk WO_3_, whereas a nitrogen-rich region is detected within the amorphous layer near the surface.

All these structural characterizations demonstrated that the WO_3_ surface was selectively nitridated while preserving the bulk crystal structure, leading to the formation of the designed heterogeneous interfacial complexion (HIC) structure—an achievement that is difficult to realize using conventional high-temperature annealing nitridation methods.^43^ First, the h-WO_3_ crystal structure represents an intermediate phase within the WO_3_ family. At elevated temperatures, around 400 °C, h-WO_3_ transitions into monoclinic WO_3_ (m-WO_3_), the most thermodynamically stable form of WO_3_.^44,45^ In addition, even when using m-WO_3_ as a starting material for nitridation, a significant degradation of its crystal structure at 500 °C occurs—this is the temperature at which WO_*x*_N_*y*_/WN begins to emerge.^43,46^ These findings highlight a key advantage of the nonequilibrium plasma nitridation approach over traditional thermal nitridation methods,^47^ i.e., its ability to achieve surface nitridation while preserving the underlying WO_3_ crystal structure, which is crucial for maintaining proton intercalation and electronic transport properties.

To gain insight into the composition and electronic structure of the amorphous WO_*x*_N_*y*_ surface layer of the HIC structure, we performed X-ray photoelectron spectroscopy (XPS), a surface-sensitive technique. As expected, there is no detectable N in the as-prepared h-WO_3_ sample, as shown in Figure 2a. After plasma-assisted surface nitridation, a distinct nitrogen signal is detected on the surface of all samples, further confirming the successful incorporation of nitrogen into the WO_3_ nanostructures. However, high-resolution XPS analysis (Figure 2b), as described below, reveals that none of the samples exhibit W^3+^-related peaks, indicating that the nitridation conditions did not lead to the formation of WN domains. This suggests that the nitridated surface likely consists of WO_*x*_N_*y*_ rather than WN.^43^ As shown in Table S1, the surface composition was further quantified based on the integrated peak areas in their respected XPS spectrum. The WO_3_-H_2_/N_2_-2h sample exhibits the highest nitrogen content of 14.3 at%, which remains significantly lower than that of any known tungsten oxynitride phases.^48^ This suggests that the amorphous HIC layer observed in Figure 1d is not composed solely of a uniform WO_*x*_N_*y*_ phase. Instead, it likely consists of a heterogeneous mixture of WO_3_ and WO_*x*_N_*y*_ domains, as schematically illustrated in Scheme 1.

**Figure 2:**
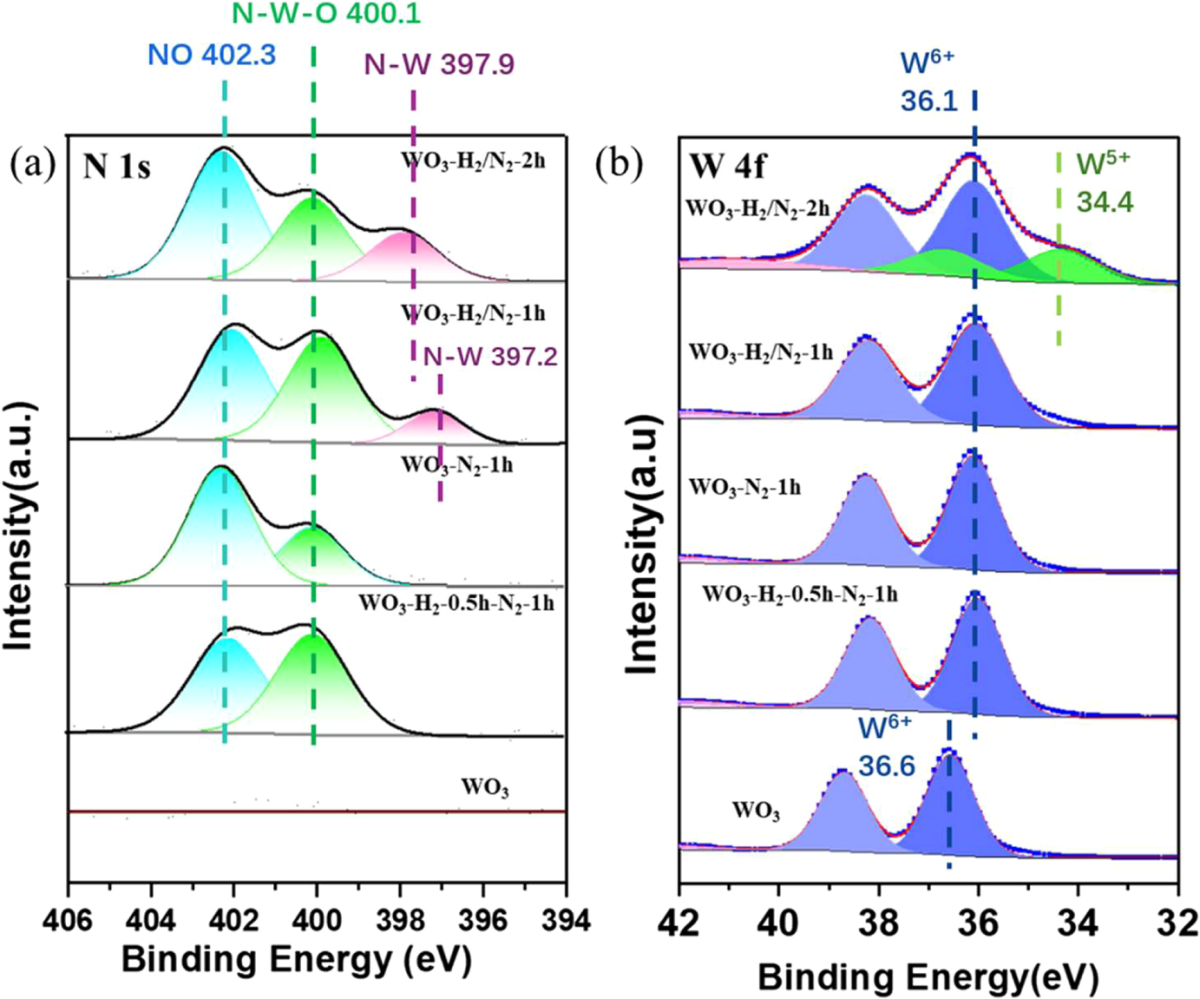
High-resolution XPS spectra of h-WO_3_ before and after plasma nitridation under different conditions: (a) N 1s, (b) W 4f.

To carefully study the chemical configuration of the N in the HIC structures, a high-resolution N 1s spectrum was collected, and the N peaks were carefully deconvoluted. As shown in Figure 2a, the high-resolution N 1s peaks for WO_3_-H_2_/N_2_-2h and WO_3_-H_2_/N_2_-1h samples can be deconvoluted into three distinct peaks, whereas for the WO_3_-N_2_-1h and WO_3_-H_2_-0.5h-N_2_-1h samples, only two peaks were observed. Based on the previous literature, the peaks in the range of 397.9–397.0 and 402.3 eV are assigned to W–N and N–O bonds, respectively.^43^ The assignment for the peak at 400.1 eV was inconsistent across the studies. Some reports assigned it to the N–W–O bond or to a nitrogen vacancy (Nv)-related bond.^43,49,50^ Nevertheless, the presence of the W–N peak suggests the successful formation of an oxynitride species. Notably, this peak appears only in samples treated with an H_2_/N_2_ plasma and is absent in N_2_ plasma-treated samples. Additionally, sequential H_2_ plasma treatment followed by N_2_ plasma treatment did not produce the same effect, suggesting that NH_*x*_ radicals formed in the H_2_/N_2_ plasma may play a role in accelerating surface nitridation.^51,52^ Furthermore, the intensity of the W–N peak in the WO_3_-H_2_/N_2_-1h sample is significantly lower than that in the WO_3_-H_2_/N_2_-2h sample, indicating that longer treatment times facilitate more N–W bond formation. Despite this, all the samples show high peak densities corresponding to N–O bonds and N–W–O/Nv-related bonds. Interestingly, the peak associated with N–W–O/Nv-related bonds in the WO_3_-H_2_/N_2_-1h sample is even higher than that in the WO_3_-H_2_/N_2_-2h sample. These results suggest a plasma nitridation pathway in which N–O and N–W–O/Nv-related species serve as intermediates in the formation of N–W bonds.

The high-resolution W 4f spectrum shows characteristic peaks at 38.8 and 36.6 eV, which correspond to W^6+^ in the as-prepared WO_3_ sample (Figure 2b, bottom curve). After plasma-assisted surface nitridation, a negative shift of approximately 0.5 eV was observed for the WO_3_-H_2_/N_2_-2h, WO_3_-H_2_/N_2_-1h, and WO_3_-N_2_-1h samples, indicating a slight reduction in the oxidation state of W upon nitridation, which is consistent with the literature.^13,43^ Two additional peaks at even lower binding energy (36.6 and 34.4 eV) were observed for the WO_3_-H_2_/N_2_-2h sample, which can be assigned to W^5+^ species.^13,53^ These results suggest that the electron density of the W centers in the nitridated samples is higher than that in the parent h-WO_3_, with WO_3_-H_2_/N_2_-2h exhibiting the highest electron density.^53^ It is reported that a higher electron density of the W centers facilities the formation of W–H* species.^33^ As we proposed, the facile formation of W–H* species plays a crucial role in promoting N hydrogenation, facilitating the formation of nitrogen vacancies (Nv) in the catalyst and accelerating the eNRR catalytic cycles in WO_*x*_N_*y*_-based catalysts (Scheme 1). Furthermore, the coexistence of WO_3_ domains and the unique HIC architecture of the catalyst is expected to sustain proton intercalation, ensuring the continuous generation and delivery of active H* species to the WO_*x*_N_*y*_ regions for the formation of W–H* (Scheme 1a). As a result, significantly enhanced eNRR activity is anticipated.

We then evaluated the eNRR performance of the WO_*x*_N_*y*_/WO_3_ hybrid catalyst electrodes and investigated the correlation between their structural characteristics and catalytic activity. The eNRR was conducted in an acidic electrolyte (H_2_SO_4_, pH = 2) using a custom-designed N_2_ flow electrolysis cell, as shown in Scheme S1a. In this configuration, the WO_*x*_N_*y*_/WO_3_ hybrid catalysts were directly employed as gas diffusion electrodes (GDEs) to address the low solubility of N_2_ in water (0.71 mg/L).^54^ The electrolyte in the working electrode (WE) side of the cell was collected for quantification of ammonium (NH_4_^+^) and possible hydrazine (N_2_H_4_) byproduct using the indophenol blue UV–vis spectroscopy method, the Watt-Chrisp method, and NMR spectroscopy as detailed in Sections S6–S8. The NH_3_ yield rate and Faradaic efficiency (FE) for the nitrogen-ammonia conversion were calculated as detailed in Section S9.

We first studied the NH_3_ yield rate and Faradaic efficiency (FE) of the WO_3_-H_2_/N_2_-2h sample, which contains the highest W–N, at different cathodic potentials. As shown in Figure S3, as the cathodic potential becomes more negative from −0.05 V to −0.25 V vs RHE, the steady-state electrolysis current increased, while the NH_3_ yield rate initially increased and then decreased, peaking at −0.15 V vs RHE. However, the FE dropped from 56.6% at −0.05 V to 40.5% and then drastically decreased to 12.2% at −0.25 V vs RHE. We attribute this drastic FE decrease to increased HER competition, as indicated by current fluctuations in the chronoamperometry curve for *t* > 1300 s, caused by H_2_ gas bubbles that accumulate and then detach from the electrode surface (Figure S3b).^12^ Based on these results, we compared the NH_3_ yield rate and FE of other catalysts at −0.15 V vs RHE. It is also worth noting that the NH_3_ yield rates were calculated based on the geometric area of catalyst electrodes, as their electrochemical surface areas (ECSAs), estimated via the double-layer capacitance (C_dl_) method, were similar (detailed in Section S10 and Figure S4). The NH_3_ yield rate and FE of WO_3_-H_2_/N_2_-1h are lower than those of WO_3_-H_2_/N_2_-2h, which can be attributed to its reduced W–N content and the absence of W^5+^ species, as confirmed by the N 1s and W 4f XPS spectra. Similarly, the significantly lower performance of WO_3_-N_2_-1h and WO_3_-H_2_-0.5h-N_2_-1h is due to the lack of W–N bonds. Among all the samples, WO_3_-H_2_/N_2_-2h demonstrates the highest NH_3_ yield rate of 3.2 × 10^–10^ mol·cm^–2^·s^–1^, accompanied by a FE of 40.1%. This enhanced performance correlates with the fact that WO_3_-H_2_/N_2_-2h contains the highest number of W–N species and exhibits the highest electron density at the W centers, both of which facilitate the formation of W–H* species, as discussed previously. Notably, this FE surpasses all previously reported values for transition metal nitride- and oxynitride-based catalysts in aqueous electrolytes, which typically exhibit FE values below 15% (Figure 3b).^17,21,22,37–41^ The remarkably high FE of the WO_*x*_N_*y*_/WO_3_ catalysts indicates significantly lower HER competition. This is especially worth mentioning because HER is generally more dominant in acidic electrolytes compared to alkaline electrolytes.^55–57^

**Figure 3:**
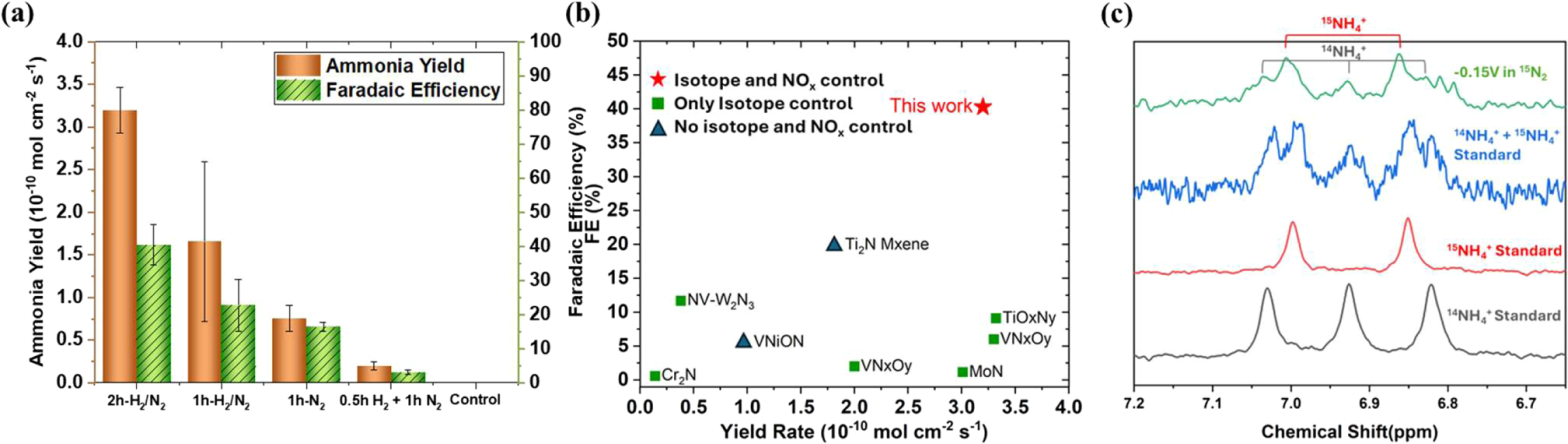
(a) Comparison of the eNRR performance of WO_3_ with different plasma treatments under identical conditions (at −0.15 V vs RHE) in the first half hour. (b) Comparison of eNRR performance of TMN- and TMO_*x*_N_*y*_-based catalysts. (c) ^1^H nuclear magnetic resonance (NMR) spectra of the electrolyte after eNRR electrolysis on WO_3_-H_2_/N_2_-2h under ^15^N_2_ purging.

To verify that the detected ammonia originates exclusively from electrocatalytic N_2_ reduction—and not from false contributions such as NH_3_ or NH_4_^+^ released from the carbon cloth (CC), electrolysis cell, electrolyte contamination, or the N_2_ gas feed—two carefully designed control experiments were performed: (1) The direct use of the h-WO_3_ nanosheet array grown on carbon cloth via the microwave hydrothermal method (without subsequent plasma nitridation) as the working electrode to perform electrolysis at −0.15 V versus RHE under N_2_ flow; and (2) the use of the WO_3_-H_2_/N_2_-2h as the working electrode to run electrolysis under N_2_ flow while the potential is kept at open circuit potential (OCP). In both cases, no detectable NH_4_^+^ was produced as shown in Figures 3a and S5a. These results not only demonstrate that h-WO_3_ alone is inactive for eNRR but also confirm that ammonia synthesis is exclusively driven by the electrochemical activity of the WO_*x*_N_*y*_/ WO_3_ catalyst.

It has been reported that commercial N_2_ gas may contain non-negligible amounts of NO_*x*_ and other impurities, which can be more readily catalytically reduced to NH_3_, potentially leading to false positive results in NH_3_ synthesis experiments.^22^ To ensure that NO_*x*_ impurities in the N_2_ feed did not contribute to the observed results, we followed the protocol developed by MacFarlane et al. to remove the possible NO_*x*_ in the N_2_ feed^58^ as illustrated in Scheme S1b. Specifically, prior to being introduced onto the back side of the GDE working electrode, the feed N_2_ gas was passed through three purification traps to effectively eliminate any NO_*x*_ impurities in the gas feed: (1) a KMnO_4_ oxidation trap to oxidize any NO_*x*_ present in the N_2_ gas into soluble NO_3_^–^, (2) a KOH trap to remove the possibly formed NO_3_^–^, and (3) a 0.1 M H_2_SO_4_ solution to trap any residual NH_3_ in the N_2_ gas. As shown in Figure S5b, the NH_3_ yield obtained after NO_*x*_ purification was approximately 8% lower than the yield achieved by using only the NH_3_ trap without NO_*x*_ purification. This difference falls within the experimental error range, confirming that under the conditions employed in this study the observed NH_3_ yield is genuine and not significantly affected by possible NO_*x*_ impurities in the N_2_ feed. Additionally, no hydrazine (N_2_H_4_) was detected in the postelectrolysis electrolyte, as shown in Figure S6.

To further confirm that the detected NH_4_^+^ originates from the electrocatalytic reduction of the feed N_2_ via the MvK mechanism, a ^15^N isotopic labeling experiment was conducted using ^15^N_2_ as the feed gas (Section S8 and Scheme S2). The resulting product was analyzed via ^1^H NMR spectroscopy, taking advantage of the distinct characteristic features between ^15^NH_4_^+^ and ^14^NH_4_^+^ in NMR spectra to verify the nitrogen source of the NH_4_^+^ product. As shown in Figure 3c, the ^1^H NMR spectrum of the electrolyte after eNRR electrolysis using the WO_3_-H_2_/N_2_-2h catalyst with ^15^N_2_ as the gas feed (top curve) displays five peaks. This spectrum closely matches that of a standard sample containing both ^15^NH_4_^+^ and ^14^NH_4_^+^. The two characteristic peaks at δ = 6.85 and 6.99 ppm are attributed to ^15^NH_4_^+^, while the three peaks at δ = 6.82, 6.93, and 7.03 ppm correspond to ^14^NH_4_^+^. The peak height for ^15^NH_4_^+^ peaks is much higher than those of ^14^NH_4_^+^ suggested that most of the detected NH_4_^+^ comes from electrocatalytic reduction of the ^15^N_2_ feed.

The simultaneous detection of ^15^NH_4_^+^ and a small amount of ^14^NH_4_^+^ in ^15^N isotope labeling experiments has been used as an indication of the MvK mechanism as both lattice nitrogen on the catalyst surface and the N_2_ feed contribute to nitrogen turnover during eNRR.^17^ This is because the initial NH_3_ generation originates from the hydrogenation of the lattice nitrogen on TMN- and TMO_*x*_N_*y*_-based electrocatalysts. This suggests that NH_3_ can be produced at the start of electrolysis without the need for an external N_2_ feed, distinguishing this process from direct proton-coupled electron transfer (PCET) of adsorbate nitrogen.^34^ However, it is important to note that TMN- and TMO_*x*_N_*y*_-based electrocatalysts can also generate NH_3_ without an external N_2_ feed through electrochemically driven decomposition, rather than by hydrogenation of surface lattice nitrogen under acidic conditions.^2^ It has been reported that electrochemical-driven decomposition of TMN has led to false positives in discovering new electrocatalysts for eNRR.^22,59–61^ Therefore, the detection of ^14^NH_4_^+^ in NMR does not yet confirm that the eNRR proceeds via the MvK mechanism. Further evidence is required to demonstrate that ^14^NH_4_^+^ is not a result of the electrochemically driven decomposition of the catalyst in acidic electrolytes.

To determine whether the detected ^14^NH_4_^+^ originated from the hydrogenation of surface lattice nitrogen or from the electrochemically driven decomposition of the catalyst in acidic electrolytes—and thereby unambiguously confirm that the WO_*x*_N_*y*_/WO_3_ catalyst follows the MvK mechanism during eNRR—a detailed XPS study of the catalyst after electrolysis was conducted. If the detected NH_3_ was due to decomposition, a concurrent loss of N and W on the WO_*x*_N_*y*_ surface would be expected.^2^ On the other hand, if the detected ^14^NH_4_^+^ was originated from hydrogenation of the surface lattice N, no W loss should be observed. As shown in Figures 4 and S7, after electrolysis, W^5+^ in the catalyst was converted to W^6+^, while the total W signal remained nearly unchanged, indicating minimal decomposition. This suggests that the ^14^NH_4_^+^ detected by NMR under N_2_ purging originates from the initial hydrogenation of lattice nitrogen in WO_*x*_N_*y*_ rather than its decomposition. Taken together, all of the experimental results confirm that the eNRR facilitated by the WO_3_-H_2_/N_2_-2h catalyst follows a MvK mechanism. In this work, we refer to it as the HIC-enhanced MvK mechanism, owing to the unique structure and properties of the heterogeneous interfacial complexion (HIC) in the catalyst.

**Figure 4:**
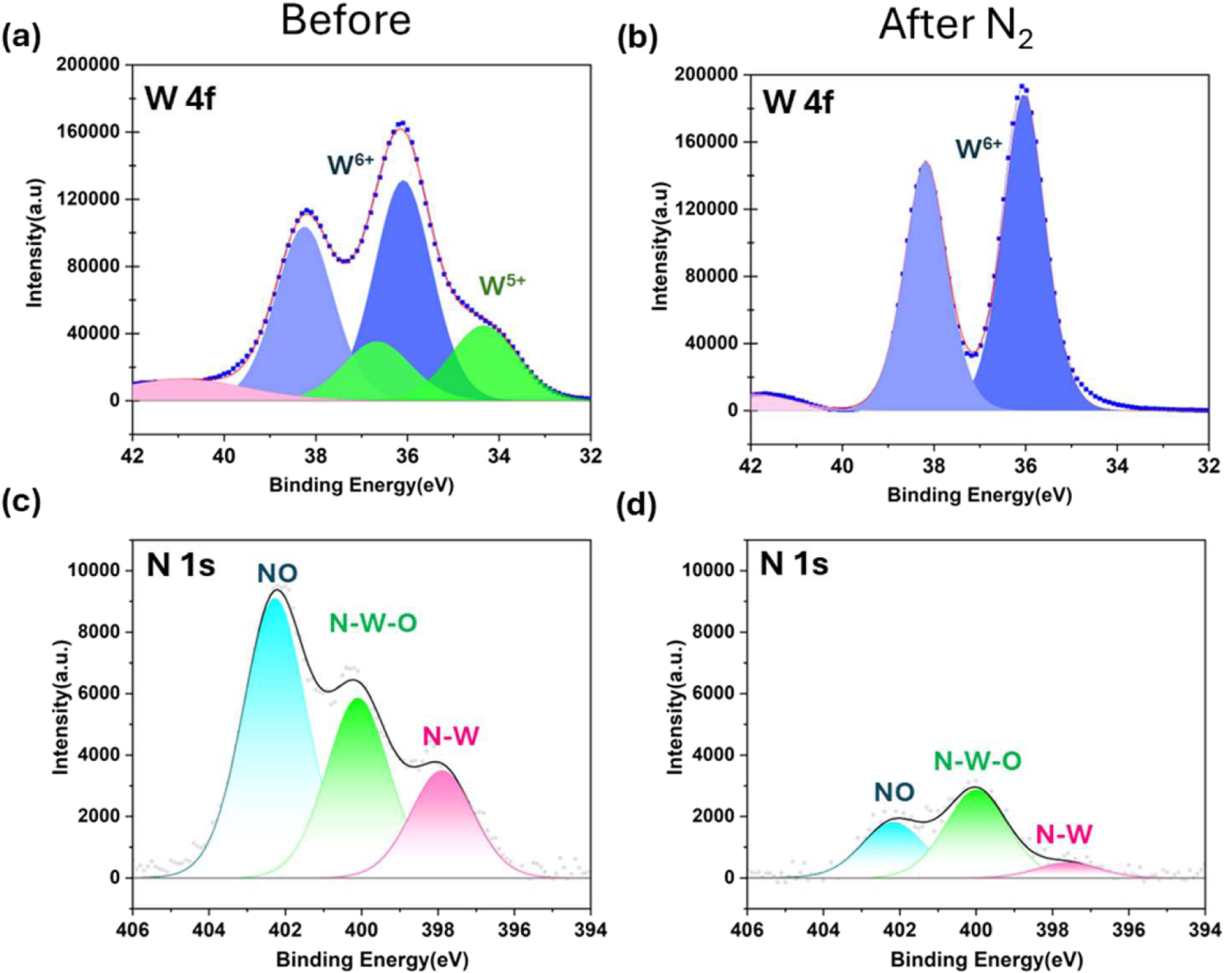
High-resolution XPS spectra of the WO_*x*_N_*y*_/WO_3_ catalyst before (a,c) and after (b,d) eNRR electrolysis for 3 h in 5 mM H_2_SO_4_ electrolyte (pH = 2) under N_2_.

We also noticed that after electrolysis, the total N content decreased and the N–W and N–O species decreased more than that of the N–W–O species (Figure 4). The decrease of the N species is consistent with the observation of decreased NH_3_ yield rate with extended electrolysis (Figure S8), which is commonly observed in TMN/TMN_*x*_O_*y*_-based eNRR.^17^ While the exact cause of the decreased NH_3_ yield rate remains under investigation, the significant decrease in N–W species may suggest that refilling the nitrogen vacancies (Nv) during eNRR may be still a limiting factor.^15,18,19^ We hypothesize that a hybrid electrochemical and plasma system with *in situ* plasma activation of N_2_ would tackle this challenge, which is currently under intensive study currently.

In summary, this work developed a hybrid proton-intercalatable WO_*x*_N_*y*_/WO_3_ electrochemical catalyst featuring a unique heterogeneous interfacial complexion (HIC) structure. This design enables facile *in situ* electrochemical generation of active hydrogen atoms (H*) in acidic electrolytes. The generated H* facilitates both nitrogen hydrogenation and nitrogen vacancy (Nv) formation, enhancing the selectivity of the electrochemical nitrogen reduction reaction (eNRR) for ammonia synthesis while effectively suppressing the competing hydrogen evolution reaction (HER). A two-step fabrication process, combining microwave-assisted hydrothermal growth with plasma-assisted surface nitridation, was developed to synthesize the WO_*x*_N_*y*_/WO_3_ heterogeneous interfacial catalyst. N_2_/H_2_ plasma was found to be more effective for surface nitridation than N_2_ plasma alone or sequential H_2_ and N_2_ treatments, likely due to the formation of reactive NH_*x*_ radicals.

The HIC-structured catalyst achieved a peak NH_3_ yield rate of 3.2 × 10^–10^ mol·cm^–2^·s^–1^ with a Faradaic efficiency of 40.1%, outperforming most TMN/TMO_*x*_N_*y*_ electrocatalysts, which typically exhibit FE < 15%. Isotopic labeling and controlled experiments confirmed that the enhanced yield and FE originated from eNRR catalyzed by the WO_*x*_N_*y*_/WO_3_ catalyst, proceeding via a MvK mechanism—referred to in this work as HIC-enhanced MvK mechanism. Notably, the NH_3_ yield rate was eight times higher and the FE approximately four times higher than that of a single-layer 2D W_2_N_3_ catalyst, despite the latter having significantly more accessible catalytic sites.^20^ This suggests significant room for further performance improvements with thinner WO_*x*_N_*y*_/WO_3_ nanosheets. Overall, this work advances the design and controllable fabrication of TMO/TMO_*x*_N_*y*_-based catalysts for enhanced eNRR activity and HER suppression.

## Supplementary Material



## Notes

Detailed information includes chemicals, experimental procedures for the fabrication and characterization of the catalysts, eNRR electrolysis setups including removing all possible contaminations in the N_2_ feed and a novel setup for economic use of the expensive ^15^N_2_ gas in the ^15^N isotope experiments, quantification of NH_4_^+^ via the indophenol blue method and NMR methods, and quantification of N_2_H_4_ via the Watt-Chrisp method (PDF)

## References

[1] Shetty A. U., Sankannavar R. (2024). Exploring
nitrogen reduction reaction mechanisms in
electrocatalytic ammonia synthesis: A comprehensive review. Journal of Energy Chemistry.

[2] Peng J., Giner-Sanz J. J., Giordano L., Mounfield W. P., Leverick G. M., Yu Y., Román-Leshkov Y., Shao-Horn Y. (2023). Design principles for transition
metal nitride stability
and ammonia generation in acid. Joule.

[3] Smith C., Hill A. K., Torrente-Murciano L. (2020). Current and future role of Haber–Bosch
ammonia in a carbon-free energy landscape. Energy
Environ. Sci..

[4] Iriawan H., Andersen S. Z., Zhang X., Comer B. M., Barrio J., Chen P., Medford A. J., Stephens I. E. L., Chorkendorff I., Shao-Horn Y. (2021). Methods for nitrogen activation by reduction and oxidation. Nature Reviews Methods Primers.

[5] Fernández C. A., Chapman O., Brown M. A., Alvarez-Pugliese C. E., Hatzell M. C. (2024). Achieving Decentralized, Electrified, and Decarbonized
Ammonia Production. Environ. Sci. Technol..

[6] Hatzell M. C. (2024). The Colors
of Ammonia. ACS Energy Letters.

[7] Gorky F., Lucero J. M., Crawford J. M., Blake B. A., Guthrie S. R., Carreon M. A., Carreon M. L. (2021). Insights
on cold plasma ammonia synthesis
and decomposition using alkaline earth metal-based perovskites. Catalysis Science & Technology.

[8] Mehta P., Barboun P., Herrera F. A., Kim J., Rumbach P., Go D. B., Hicks J. C., Schneider W. F. (2018). Overcoming
ammonia synthesis scaling relations with plasma-enabled catalysis. Nature Catalysis.

[9] Ju, Y. ; Starikovskiy, A. Plasma Assisted Combustion and Chemical Processing; CRC press, 2025.

[10] Qu Z., Zhou R., Sun J., Gao Y., Li Z., Zhang T., Zhou R., Liu D., Tu X., Cullen P. (2024). Plasma-Assisted Sustainable Nitrogen-to-Ammonia
Fixation:
Mixed-phase, Synergistic Processes and Mechanisms. ChemSusChem.

[11] Sun J., Alam D., Daiyan R., Masood H., Zhang T., Zhou R., Cullen P. J., Lovell E. C., Jalili A., Amal R. (2021). A hybrid plasma electrocatalytic
process for sustainable ammonia
production. Energy Environ. Sci..

[12] Zhang T., Zhou R., Zhang S., Zhou R., Ding J., Li F., Hong J., Dou L., Shao T., Murphy A. B. (2023). Sustainable Ammonia
Synthesis from Nitrogen and Water by One-Step
Plasma Catalysis. Energy & Environmental
Materials.

[13] Li Q., Kucukosman O. K., Ma Q., Ouyang J., Kucheryavy P., Gu H., Long C. L., Zhang Z., Young J., Lockard J. V. (2024). Enhancement of Electrochemical Nitrogen Reduction Activity and Suppression
of Hydrogen Evolution Reaction for Transition Metal Oxide Catalysts:
The Role of Proton Intercalation and Heteroatom Doping. ACS Catal..

[14] Suryanto B. H. R., Du H.-L., Wang D., Chen J., Simonov A. N., MacFarlane D. R. (2019). Challenges
and prospects in the catalysis of electroreduction
of nitrogen to ammonia. Nature Catalysis.

[15] Deng J., Iniguez J. A., Liu C. (2018). Electrocatalytic Nitrogen Reduction
at Low Temperature. Joule.

[16] Abghoui Y., Skulason E. (2017). Electrochemical synthesis
of ammonia via Mars-van Krevelen
mechanism on the (111) facets of group III-VII transition metal mononitrides. Catal. Today.

[17] Yang X., Nash J., Anibal J., Dunwell M., Kattel S., Stavitski E., Attenkofer K., Chen J. G., Yan Y., Xu B. (2018). Mechanistic Insights
into Electrochemical Nitrogen Reduction Reaction
on Vanadium Nitride Nanoparticles. J. Am. Chem.
Soc..

[18] Ologunagba D., Kattel S. (2021). Transition metal oxynitride catalysts for electrochemical
reduction of nitrogen to ammonia. Materials
Advances.

[19] Abghoui Y., Skúlason E. (2017). Computational Predictions of Catalytic Activity of
Zincblende (110) Surfaces of Metal Nitrides for Electrochemical Ammonia
Synthesis. J. Phys. Chem. C.

[20] Abghoui Y., Garden A. L., Howalt J. G., Vegge T., Skúlason E. (2016). Electroreduction
of N2 to Ammonia at Ambient Conditions on Mononitrides of Zr, Nb,
Cr, and V: A DFT Guide for Experiments. ACS
Catal..

[21] Jin H., Li L., Liu X., Tang C., Xu W., Chen S., Song L., Zheng Y., Qiao S.-Z. (2019). Nitrogen
Vacancies
on 2D Layered W_2_N_3_: A Stable and Efficient Active
Site for Nitrogen Reduction Reaction. Adv. Mater..

[22] Young S. D., Ceballos B. M., Banerjee A., Mukundan R., Pilania G., Goldsmith B. R. (2022). Metal Oxynitrides for the Electrocatalytic Reduction
of Nitrogen to Ammonia. J. Phys. Chem. C.

[23] Osonkie A., Ganesan A., Chukwunenye P., Anwar F., Balogun K., Gharaee M., Rashed I., Cundari T. R., D’Souza F., Kelber J. A. (2022). Electrocatalytic Reduction of Nitrogen to Ammonia:
the Roles of Lattice O and N in Reduction at Vanadium Oxynitride Surfaces. ACS Applied Materials & Interfaces.

[24] Cui L., Sun Z., Wang Y., Jian X., Li H., Zhang X., Gao X., Li R., Liu J. (2024). *H migration-assisted MvK mechanism
for efficient electrochemical NH_3_ synthesis over TM–TiNO. Phys. Chem. Chem. Phys..

[25] Yang X. J., Xu B. J., Chen J. G. G., Yang X. (2023). Recent Progress in
Electrochemical Nitrogen Reduction on Transition Metal Nitrides. ChemSusChem.

[26] Ye T. N., Park S. W., Lu Y., Li J., Sasase M., Kitano M., Hosono H. (2020). Contribution of Nitrogen
Vacancies
to Ammonia Synthesis over Metal Nitride Catalysts. J. Am. Chem. Soc..

[27] Ye T.-N., Park S.-W., Lu Y., Li J., Sasase M., Kitano M., Tada T., Hosono H. (2020). Vacancy-enabled
N_2_ activation for ammonia synthesis on an Ni-loaded catalyst. Nature.

[28] Zhang Z., Feng X., Zhang Z., Chen L., Liu W., Tong L., Gao X., Zhang J. (2024). Graphdiyne Enabled
Nitrogen Vacancy Formation in Copper Nitride for Efficient Ammonia
Synthesis. J. Am. Chem. Soc..

[29] Dillon S. J., Tang M., Carter W. C., Harmer M. P. (2007). Complexion: A new
concept for kinetic engineering in materials science. Acta Mater..

[30] Kiafiroozkoohi N. S., Ghorbani S. R., Arabi H., Ghanbari R. (2024). Electrochemical performance
engineering of tungsten oxynitride by a nitrogen controlling synthesis
method for a binder-free supercapacitor electrode. Journal of Energy Storage.

[31] Chen W. P., He K. F., Wang Y., Chan H. L. W., Yan Z. (2013). Highly mobile
and reactive state of hydrogen in metal oxide semiconductors at room
temperature. Sci. Rep..

[32] Yan B., Bisbey R. P., Alabugin A., Surendranath Y. (2019). Mixed Electron–Proton
Conductors Enable Spatial Separation of Bond Activation and Charge
Transfer in Electrocatalysis. J. Am. Chem. Soc..

[33] Miu E. V., McKone J. R., Mpourmpakis G. (2022). The Sensitivity of Metal Oxide Electrocatalysis
to Bulk Hydrogen Intercalation: Hydrogen Evolution on Tungsten Oxide. J. Am. Chem. Soc..

[34] Ren Y., Yu C., Tan X., Wei Q., Wang Z., Ni L., Wang L., Qiu J. (2022). Strategies
to activate inert nitrogen
molecules for efficient ammonia electrosynthesis: current status,
challenges, and perspectives. Energy Environ.
Sci..

[35] Ling C., Zhang Y., Li Q., Bai X., Shi L., Wang J. (2019). New Mechanism for N_2_ Reduction: The Essential Role of
Surface Hydrogenation. J. Am. Chem. Soc..

[36] Feng X., Liu J., Chen L., Kong Y., Zhang Z., Zhang Z., Wang D., Liu W., Li S., Tong L. (2023). Hydrogen Radical-Induced Electrocatalytic N_2_ Reduction
at a Low Potential. J. Am. Chem. Soc..

[37] Zhou Y. Y., Fu X. B., Chorkendorff I., Norskov J. K. (2025). Electrochemical
Ammonia Synthesis: The Energy Efficiency Challenge. ACS Energy Letters.

[38] Johnson D., Hunter B., Christie J., King C., Kelley E., Djire A. (2022). Ti_2_N nitride
MXene evokes the Mars-van Krevelen mechanism
to achieve high selectivity for nitrogen reduction reaction. Sci. Rep..

[39] Nash J., Yang X., Anibal J., Dunwell M., Yao S., Attenkofer K., Chen J. G., Yan Y., Xu B. (2019). Elucidation
of the Active Phase and Deactivation Mechanisms of Chromium Nitride
in the Electrochemical Nitrogen Reduction Reaction. J. Phys. Chem. C.

[40] Chang B., Deng L., Wang S., Shi D., Ai Z., Jiang H., Shao Y., Zhang L., Shen J., Wu Y. (2020). A vanadium–nickel
oxynitride layer for enhanced
electrocatalytic nitrogen fixation in neutral media. Journal of Materials Chemistry A.

[41] Kang S., Wang J., Zhang S., Zhao C., Wang G., Cai W., Zhang H. (2019). Plasma-etching
enhanced titanium oxynitride active
phase with high oxygen content for ambient electrosynthesis of ammonia. Electrochem. Commun..

[42] Gao L., Wang X., Xie Z., Song W., Wang L., Wu X., Qu F., Chen D., Shen G. (2013). High-performance energy-storage
devices based on WO_3_ nanowire arrays/carbon cloth integrated
electrodes. Journal of Materials Chemistry A.

[43] Huang Z., Yang B., Zhou Y., Luo W., Chen G., Liu M., Liu X., Ma R., Zhang N. (2023). Tungsten Nitride/Tungsten
Oxide Nanosheets for Enhanced Oxynitride Intermediate Adsorption and
Hydrogenation in Nitrate Electroreduction to Ammonia. ACS Nano.

[44] Chen Z., Peng Y. T., Liu F., Le Z. Y., Zhu J., Shen G. R., Zhang D. Q., Wen M. C., Xiao S. N., Liu C. P. (2015). Hierarchical
Nanostructured WO_3_ with Biomimetic
Proton Channels and Mixed Ionic-Electronic Conductivity for Electrochemical
Energy Storage. Nano Lett..

[45] Kalanur S. S., Hwang Y. J., Chae S. Y., Joo O. S. (2013). Facile growth of
aligned WO_3_ nanorods on FTO substrate for enhanced photoanodic
water oxidation activity. Journal of Materials
Chemistry A.

[46] Shi J., Pu Z., Liu Q., Asiri A. M., Hu J., Sun X. (2015). Tungsten nitride
nanorods array grown on carbon cloth as an efficient hydrogen evolution
cathode at all pH values. Electrochim. Acta.

[47] Dong S., Chen X., Zhang X., Cui G. (2013). Nanostructured transition
metal nitrides for energy storage and fuel cells. Coord. Chem. Rev..

[48] Zhang B., Zheng Y., Xing Z., Wu Z., Cheng C., Ma T., Li S. (2024). Interfacial electron-engineered
tungsten oxynitride
interconnected rhodium layer for highly efficient all-pH-value hydrogen
production. Journal of Materials Chemistry A.

[49] Yang X., Zhao F., Yeh Y. W., Selinsky R. S., Chen Z., Yao N., Tully C. G., Ju Y., Koel B. E. (2019). Nitrogen-plasma
treated hafnium oxyhydroxide as an efficient acid-stable electrocatalyst
for hydrogen evolution and oxidation reactions. Nat. Commun..

[50] Liu D., Xu Y., Sun M., Huang Y., Yu Y., Zhang B. (2020). Photothermally
assisted photocatalytic conversion of CO_2_–H_2_O into fuels over a WN–WO_3_ Z-scheme heterostructure. Journal of Materials Chemistry A.

[51] van
Helden J. H., van den Oever P. J., Kessels W. M. M., van
de Sanden M. C. M., Schram D. C., Engeln R. (2007). Production Mechanisms
of NH and NH_2_ Radicals in N_2_–H_2_ Plasmas. J. Phys. Chem. A.

[52] Liu J., Lu H., Zhang D. W., Nolan M. (2022). Self-limiting nitrogen/hydrogen plasma
radical chemistry in plasma-enhanced atomic layer deposition of cobalt. Nanoscale.

[53] Cong S., Yuan Y. Y., Chen Z. G., Hou J. Y., Yang M., Su Y. L., Zhang Y. Y., Li L., Li Q. W., Geng F. X. (2015). Noble metal-comparable
SERS enhancement from semiconducting
metal oxides by making oxygen vacancies. Nat.
Commun..

[54] Hu L., Xing Z., Feng X. (2020). Understanding
the Electrocatalytic
Interface for Ambient Ammonia Synthesis. ACS
Energy Letters.

[55] Gebremariam G. K., Jovanović A. Z., Pašti I. A. (2023). The Effect
of Electrolytes on the
Kinetics of the Hydrogen Evolution Reaction. Hydrogen.

[56] Chen J., Chen C., Qin M., Li B., Lin B., Mao Q., Yang H., Liu B., Wang Y. (2022). Reversible hydrogen
spillover in Ru-WO_(3‑x)_ enhances hydrogen evolution
activity in neutral pH water splitting. Nat
Commun.

[57] Subbaraman R., Tripkovic D., Strmcnik D., Chang K.-C., Uchimura M., Paulikas A. P., Stamenkovic V., Markovic N. M. (2011). Enhancing Hydrogen
Evolution Activity in Water Splitting by Tailoring Li^+^-Ni­(OH)_2_-Pt Interfaces. Science.

[58] Choi J., Suryanto B. H. R., Wang D., Du H.-L., Hodgetts R. Y., Ferrero Vallana F. M., MacFarlane D. R., Simonov A. N. (2020). Identification and
elimination of false positives in electrochemical nitrogen reduction
studies. Nat. Commun..

[59] Du H.-L., Gengenbach T. R., Hodgetts R., MacFarlane D. R., Simonov A. N. (2019). Critical Assessment
of the Electrocatalytic Activity
of Vanadium and Niobium Nitrides toward Dinitrogen Reduction to Ammonia. ACS Sustainable Chem. Eng..

[60] Manjunatha R., Karajić A., Teller H., Nicoara K., Schechter A. (2020). Electrochemical
and Chemical Instability of Vanadium Nitride in the Synthesis of Ammonia
Directly from Nitrogen. ChemCatChem..

[61] Hu B., Hu M., Seefeldt L., Liu T. L. (2019). Electrochemical Dinitrogen Reduction
to Ammonia by Mo_2_N: Catalysis or Decomposition?. ACS Energy Letters.

