# Supplementary material for: Plasma-Assisted Surface Nitridation of Proton Intercalatable WO3 for Efficient Electrocatalytic Ammonia Synthesis
Source: ACS Energy Lett. 2025 Jun 22;10(7):3349–58. doi: 10.1021/acsenergylett.5c01034 (PMC12333582; doi:10.1021/acsenergylett.5c01034)
Supplement: Supplementary file 1 [file nz5c01034_si_001.pdf]

Supporting Information for

Plasma Assisted Surface Nitridation of Proton Intercalatable WO_3 for Efficient Electrocatalytic Ammonia Synthesis

Zhiyuan Zhang [a], *Christopher Kondratowicz* [b], *Jacob Smith* [c], *Pavel Kucheryavy* [a], *Junjie Ouyang* [a], *Yijie Xu* [b], *Elizabeth Desmet* [b], *Sophia Kurdziel* [d], *Eddie Tang* [b, e], *Micheal Adeleke* [a, f], *Aditya Dilip Lele* [g], *John Mark Martirez* [d], *Miaofang Chi* [c, h], *Yiguang Ju* [b, d*], *Huixin He* [a*]

[a] Department of Chemistry, Rutgers, the State University of New Jersey, Newark, New Jersey 07102, United States

[b] Department of Mechanical and Aerospace Engineering, Princeton University, Princeton, New Jersey 08544, United States

[c] Center for Nanophase Materials Sciences, Oak Ridge National Laboratory, Oak Ridge, Tennessee 37831, United States

[d] Applied Materials and Sustainability Sciences, Princeton Plasma Physics Laboratory, Princeton, New Jersey 08543, United States

[e] Montgomery High School, Skillman, New Jersey 08558, United States

[f] Science Park High School, Newark, New Jersey 07103, United States

[g] Department of Mechanical Engineering, Rowan University, Glassboro, New Jersey 08028, United States

[h] Thomas Lord Department of Mechanical Engineering & Materials Science, Duke University, Durham, North Carolina 27708, United States

S1. Fast fabrication of WO_3 nanosheet array on carbon cloth with the microporous layer (MPL) via a microwave hydrothermal method

A microwave hydrothermal method was applied to fabricate the WO_3 nanosheet array directly on a carbon cloth support with the microporous layer (MPL). The recipe for the fabrication was slightly modified from the work by Gao *et al*[1]. In brief, 1.25 mmol of sodium tungstate dihydrate ($Na_2WO_4 \cdot 2H_2O$) was dissolved in deionized water (10 mL) under vigorous stirring for 20 min. Subsequently a 3M HCl aqueous solution was slowly dropped into the solution until the pH value of the solution reached 1.2 to form a yellowish transparent solution. Then, 3.5 mmol oxalic acid ($H_2C_2O_4$) was added into the above mixture and diluted to 25 mL, which resulted in the formation of the $H_2C_2O_4$ precursor. For the next step, the as-prepared 4 mL H_2WO_4 precursor was transferred into a microwave tube, and then 0.1 g of Na_2SO_4 was added to the solution to control the structure. A piece of carbon cloth (0.75cm × 0.75 cm in size) with the MPL (Hydro-LAT 1400, Fuel Cells ETC), which was ultrasonically cleaned by deionized water and alcohol in sequence, was put into the microwave tube and sealed, and maintained at 180 °C for 15 minutes by microwave oven (Discover SP, CEM). After the autoclave cooled down to room temperature, the WO_3 membrane was taken out and rinsed with deionized water several times and dried at 70 °C in ambient.

S2. Plasma assisted surface nitridation of the WO_3 nanosheet array

The as-fabricated WO_3 nanosheet arrays on carbon cloth support were treated with 16-torr N_2 and N_2/H_2 plasma for different durations, as specified in the sample names. The plasma was generated in a home-made DBD cell, powered by a 20-kHz, 13-kV AC

power supply. After plasma treatment, the composition, oxidation states, and morphology of the samples were analyzed by a range of surface characterization techniques, including X-ray photoelectron spectroscope (XPS), scanning electron microscope (SEM) and high-angle annular dark-field scanning transmission electron microscopy (HAADF-STEM).

S3. Characterization of the Electrocatalysts

The morphology of the WO_3 and WO_xN_y/WO_3 was analyzed by field emission scanning electron microscope (FE-SEM) (Hitachi S-4800 and JSM-7900F, JEOL, Japan) at 15 kV. The crystalline structures were investigated by X ray diffraction (XRD, a Rigaku Miniflex 6G) with a Co Ka radiation (λ = 1.789 Å). The surface chemical states and compositions were measured by X-ray photoelectron spectroscope (XPS, Thermo, K-Alpha, USA) with a monochromatic Al Kα radiation (hv = 1486.6 eV). All spectra were calibrated using the binding energy of C 1s (284.8 eV) as a reference.

High-angle annular dark-field scanning transmission electron microscopy (HAADF-STEM) was acquired on an aberration-corrected JEOL NEOARM operating at 200 kV using a convergence semi-angle of 28 mrad. Any electron energy loss spectroscopy (EELS) data was acquired on an aberration corrected FEI Titan microscope operating at 300 kV using a convergence semi-angle of 19.3 mrad. A 0.1 eV/channel dispersion was used during dual EELS data acquisition, wherein the low loss contains the zero-loss peak and the high loss contains both the O K-edge and N K-edge simultaneously. A full-width half maximum of the zero-loss peak registers an energy resolution of 1.0 eV. STEM samples were prepared using a solution-casting technique. The initial sample powders were dispersed in isopropyl alcohol and sonicated. Thereafter, the solution was applied to conventional lacey carbon TEM foil grids.

S4. Assessment of eNRR performance of the WO_xN_y/WO_3 hybrid catalyst electrodes

All the electrochemical measurements were performed using a CHI 760 C Potentiostat (CH Instruments, USA). Ag/AgCl (sat. KCl) and Pt wire were used as reference (RE) and counter electrodes (CE), respectively. The applied potentials measured against the Ag/AgCl reference electrode in saturated KCl were converted to the reversible hydrogen electrode (RHE) using the equation:

$$RHE = E_{Ag/AgCl} + 0.197\ V + 0.059 \times pH \qquad (eq1)$$

S5. eNRR setup

The eNRR performance of the WO_xN_y/WO_3 hybrid catalyst electrodes was evaluated using a CH Instruments 760E Potentiostat with a homemade designed N_2 flow electrolysis cell as shown in **Scheme S1a.** This cell consists of a proton exchange membrane (Nafion 117, Dupont), a piece of Pt plate, Ag/AgCl (saturated KCl) electrode, which act as the counter electrode (CE), and reference electrode, respectively. The working electrode (WE) is the as-prepared WO_xN_y/WO_3 hybrid catalyst electrodes with a size of 0.7 × 0.7 cm, which were directly used as a catalytic gas-diffusion electrode (GDE) for eNRR to alleviate the low solubility issue of N_2 in water-based electrolytes (0.71 mg/mL). To eliminate potential NO_x and NH_3 contamination in the N_2 gas, pressurized N_2 (flow rate: 2.5 mL/min) from the gas tank was passed through three traps arranged in series before being introduced to the backside of the GDE (the side without catalyst coating) for eNRR **(Scheme S1b)**. The three purification traps included: (1) A $KMnO_4$ oxidation trap to oxidize any NO_3 present in the N_2 gas into soluble NO_3^-. (2) A KOH trap to remove the possibly formed NO_3^-. (3) A 0.1 M H_2SO_4 solution to trap any residual NH_3 in the N_2 gas. After purification, the N_2 gas was purged onto the backside of the GDE to initiate eNRR and the excess gas was

purged back to the electrolyte to avoid the loss of the produced NH_3 carried over by the N_2 flow during the eNRR. The WE side and the CE side were separated by a Nafion 117 membrane (Fuel cell store). The electrolysis was performed for 0.5 h with constant potential in a H_2SO_4 solution (pH = 2) as the electrolyte. The electrolyte in the WE side of the cell was collected for ammonium (NH_3) and hydrazine (N_2H_4) detection using the indophenol blue UV-Vis spectroscopy method and NMR spectroscopy as detailed **in sections S6-8.** The NH_3 yield rate and the Faradic efficiency for the nitrogen-ammonia conversion were calculated as detailed in **section S9.**

S6. Determination of NH_4^+ via the indophenol blue method

The concentration of the produced NH_4^+ was spectrophotometrically determined by the indophenol blue method. Typically, 1 mL of electrolyzed electrolyte was transferred from the electrochemical cell to a clean vial. Then 1 mL of solution containing 1M NaOH with 5 wt% salicylic acid and 5 wt% sodium citrate was added to the vial. Then 0.5 mL of 5 wt% NaClO aqueous solution and 0.1 mL of 1 wt% sodium nitroprusside solution was sequentially dropwise added to the mixture. After reacting for 2 h, the UV-vis spectrum was collected from 750 nm to 500 nm. Calibration curve was made using NH_4Cl standard (BTC) diluted to 0.4, 0.8, 1.2, 1.6 ppm (NH_4^+ concentration) with the fresh electrolyte. Calibration curve was made at each time the electrolyzed electrolytes were measured.

S7. Determination of N_2H_4

N_2H_4 was determined by Watt and Chrisp colorimetric method. In brief, the color reagent was obtained by mixing concentrated HCl (30 mL), C_2H_5OH (300 mL) and $C_9H_{11}NO$ (5.99 g). And 5 mL electrolyte was taken from the cathodic chamber and added into 5 mL above as-prepared color reagent. After standing for 15 minutes, the absorption

spectrum of the solution was collected in the wavelength range of 420-500 nm, and the peak appears at 455 nm. The calibration curve was measured using the absorbance of N_2H_4 solution with different concentrations. Calibration curve displays good linear relationship of absorbance with N_2H_4 concentrations ($y = 1.0158x + 0.1519$, $R^2 = 0.9991$) by taking the mean of three times independent calibration.

S8. $^{15}N_2$ isotope label experiment and NMR Analysis:

Before starting the nitrogen reduction reaction (NRR) experiment using ^{15}N-labeled nitrogen, the system was purged with argon gas three times over a total of 30 minutes to remove any residual air. After purging, the argon supply was turned off, and the balloon was evacuated by pump before being filled with $^{15}N_2$ gas. The electrolysis was then conducted at -0.15 V vs RHE for 30 minutes. Following electrolysis, the electrolyte was collected for further analysis of the ^{15}N-labeled products.

For NMR sample preparation, the pH of the electrolyte was adjusted to 4.5, maleic acid was added as an internal standard, and DMSO-d_6 was used to lock the sample. NH_4^+ detection via NMR was performed using the excitation sculpting water suppression (zgesgp pulse sequence) on a Bruker 500 MHz Avance III HD spectrometer. To improve the signal-to-noise ratio, the following parameters were optimized: frequency offset (O1P), size of the free induction decay (FID) (TD), pre-scan delay (D1), and the number of scans (NS). While O1P varied from experiment to experiment, TD was set to 16,378, D1 to 1 s, and NS to 16,378.

Before starting the NRR experiment using ^{15}N-labeled nitrogen, the system was purged with argon gas three times over a total of 30 minutes to remove residual air. Afterward, the argon supply was shut off, and the balloon was evacuated by pump and subsequently filled with $^{15}N_2$ gas. The electrolysis was then carried out at -0.15V vs RHE for 30 minutes. Finally, the electrolyte was collected for further analysis of the ^{15}N-labeled products. NMR samples were prepared in the particular way: after electrolysis pH was adjusted to 4.5, maleic acid was added as internal standard, and DMSO-d6 to lock. NH_4^+ detection via NMR was performed using the excitation sculpting water suppression (zgesgp pulse sequence) on Bruker 500 MHz Avance III HD spectrometer. For better signal-to-noise ratio the following parameters were optimized: frequency offset (O1P), size of FID (TD), pre-scan delay (D1), and number of scans (NS). While O1P was varied from experiment-to-experiment TD was set to 16378, D1 to 1 s, and NS to 16378.

S9. Calculation of NH_3 yield rate and Faradic efficiency for ammonia production

The NH_3 yield rate was calculated by Eq. (S2)

$$R_{NH_3} = \frac{C[NH_4^+]\times V}{s_{cat}\times t} \qquad \text{(eq2)}$$

where $C[NH_4^+]$ is the concentration of $[NH_4^+]$ in the electrolyte in the WE side, V is the volume of the electrolyte, S_{cat} is the catalyst electrode area, t is the electrolysis duration.

The Faradic efficiency for the nitrogen-ammonia conversion was calculated by Eq. (S3):

$$FE = \frac{3\times C[NH_4^+]\times V\times F}{Q} \qquad \text{(eq3)}$$

where Q is the total charge passed during the electrolysis, F is Faraday constant (96485 C mol^{-1}).

S10. Estimation of electrochemical surface areas (ECSAs) of the catalysts

To ensure consistent WO_3 coverage—and thus identical surface areas—across all electrodes when assessing the impact of nitrogen content, every sample was deposited on a 0.5 cm^2 geometric area and prepared under strictly controlled microwave conditions. The resulting products were carefully characterized using SEM and the double-layer capacitance (C_{dl}) method to ensure a consistent density of WO_3 nanosheets on the carbon cloth support. The C_{dl} method was used to estimate the ECSA of the WO_3 catalysts before and after nitridation. As shown in Figure **S4**, nonpolarized cyclic voltammetry (CV) was performed in the range of 0.30–0.40 V vs RHE. The C_{dl} of each WO_3 sample before nitridation is consistently around 1.51 mF cm^{-2}, while the C_{dl} for WO_3-H_2/N_2-2h increased slightly to 2.02 mF cm^{-2}. This increase may be attributed to its higher conductivity and surface amorphization, as seen in the TEM image. Nevertheless, this minor change in C_{dl} suggests that the ECSA change is negligible compared to the significant enhancement in NRR activity. Therefore, we conclude that the surface areas of the electrodes are similar, and the observed performance differences are primarily due to nitrogen incorporation rather than surface area effects.

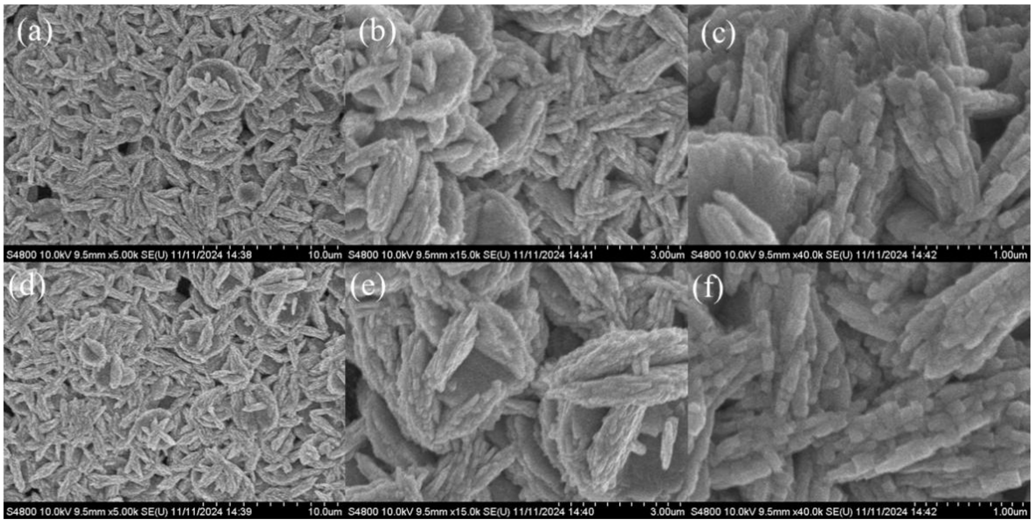

Figure S1. (a, b and c) Typical SEM images of h-WO_3 at different magnifications. (d, e and f) Typical SEM images of WO_3-H_2/N_2-2h with the same magnifications as in a-c for comparison.

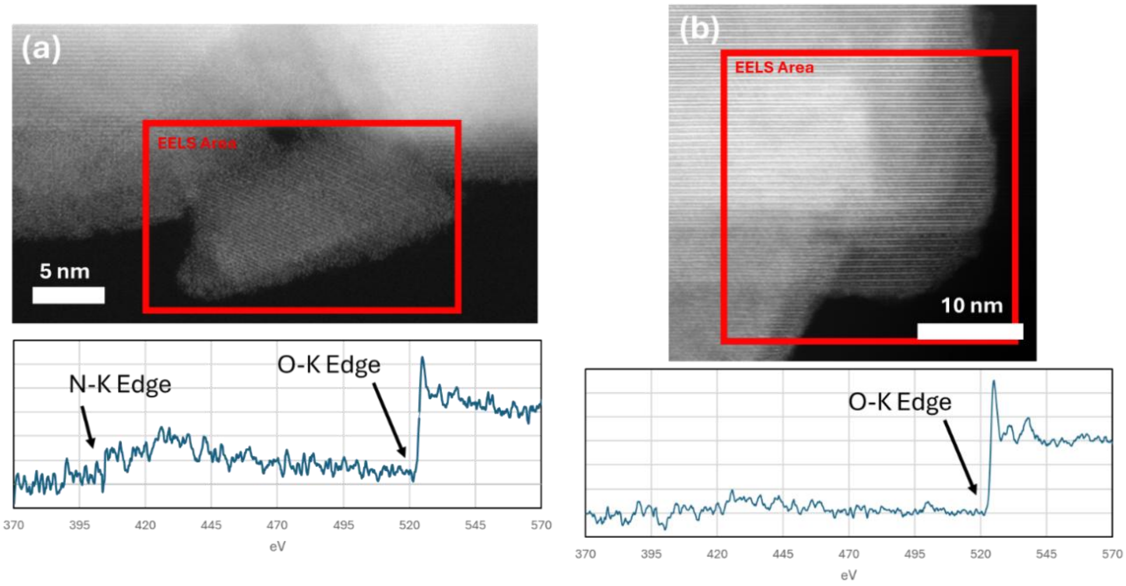

Figure S2. Observing surface nitridation of WO_3 after plasma treatment using EELS. (a) N-rich regions of WO_3 are evident by the presence of severe surface reconstruction. By comparison, (b) minimal N-signal is observed when bulk WO_3 EELS signals dominate the spectra.

Table S1. XPS quantification of the deconvoluted peaks shown in **Figure 2**.

Sample	W at%	O at%	N at%	$N_{(N\text{-}W)}/N_{total}$ (%)
WO_3-H_2/N_2-2h	25.2	60.5	14.3	19
WO_3-H_2/N_2-1h	23.5	65.5	10.8	12
WO_3-N_2-1h	23.9	68.5	7.6	0
WO_3-H_2-0.5h-N_2-1h	23.1	66.7	10.2	0

at%: Atomic Percentage

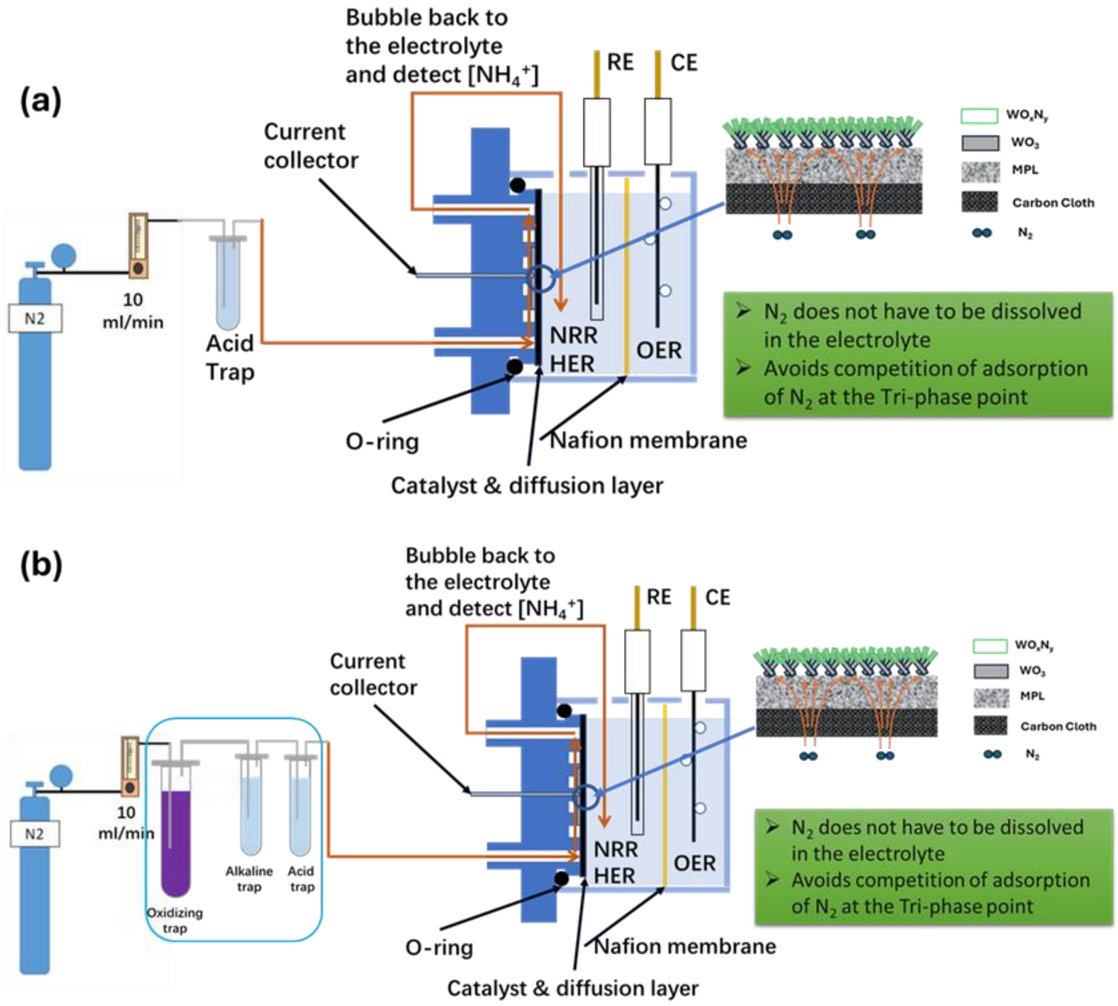

Scheme S1. Schematic drawings to show the (a) set-up for the eNRR electrolysis. (b) NO_x control set-up: any NO_x breakthrough from the oxidizing trap (filled with 0.1 M $KMnO_4$ in aqueous 0.1 M KOH) is captured by an alkaline trap (filled with aqueous 0.1 M KOH). All NH_3 breakthroughs and alkaline solutions will be captured by the acid trap (filled with concentrated H_2SO_4).

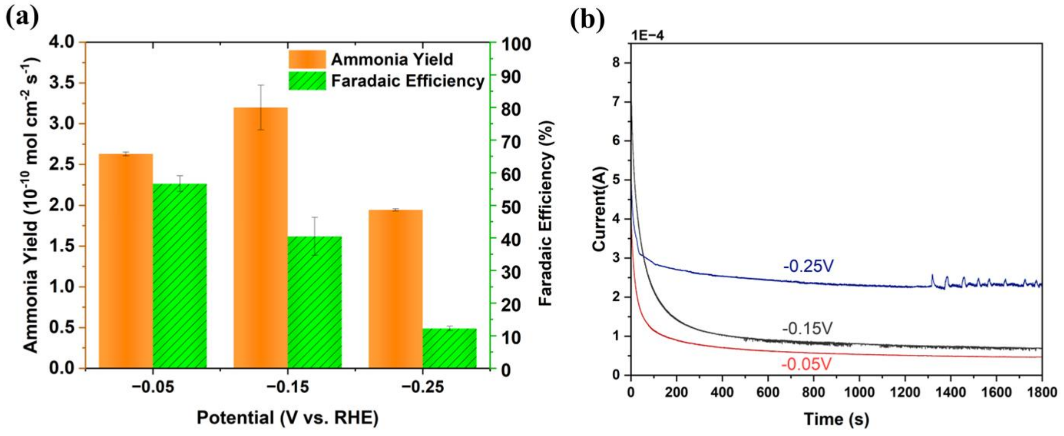

Figure S3. (a) Specific yield rate and faradaic efficiency of WO_3-H_2/N_2-2h at different cathodic potentials. (b) Chronoamperometry curves of WO_3-H_2/N_2-2h catalyst at different potentials for 30 min in pH = 2 H_2SO_4 electrolyte.

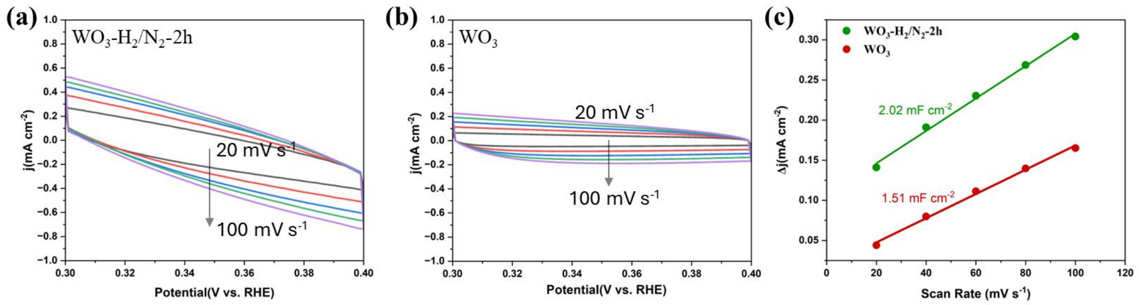

Figure S4. Cyclic voltammetry (CV) curves at the non-Faraday reaction region with different scanning rates (a) WO_3-H_2/N_2-2h, (b)WO_3. (c) The current density at 0.35V as a

function of scanning rates of the CVs. The C_{dl} of the WO_3-H_2/N_2-2h and WO_3 were estimated by the slopes.

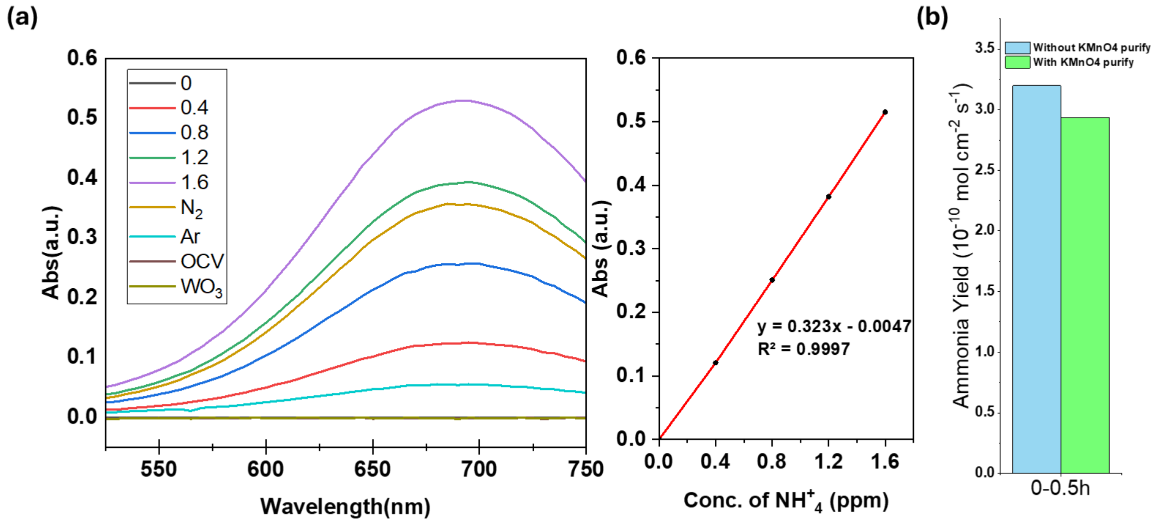

Figure S5. (a) UV–vis spectra of the electrolytes collected from the various control experiments following the indophenol blue spectrophotometric method. (b) comparison of NH_3 yield rate with/without NO_x removal from the N_2 feed.

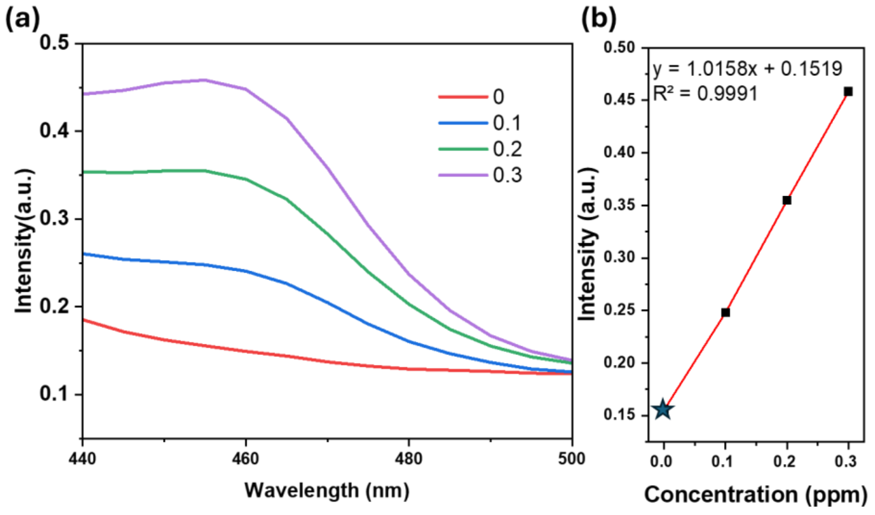

Figure S6. (a) UV-VIS spectra of various N_2H_4 concentrations after incubated for 15 min at room temperature. (b) Calibration curve used for calculation of N_2H_4 concentrations.

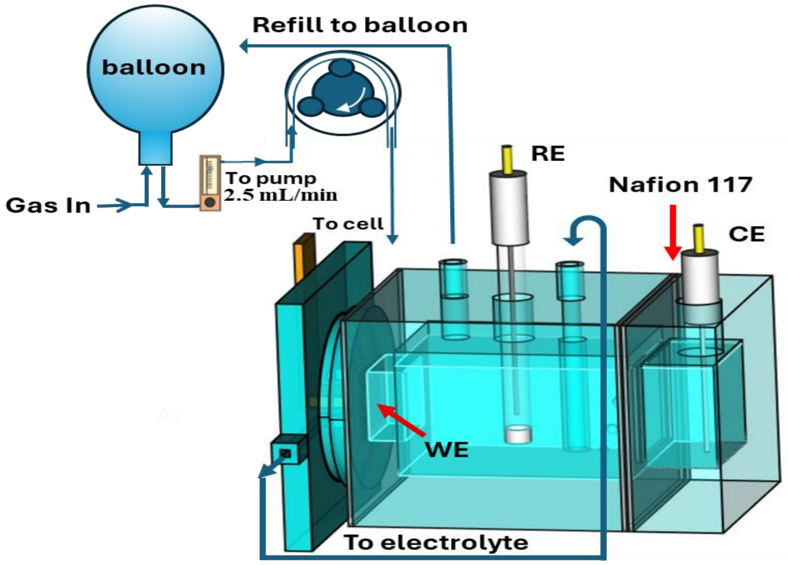

Scheme S2. Schematic drawings to show the set-up for the $^{15}N_2$ isotope eNRR electrolysis.

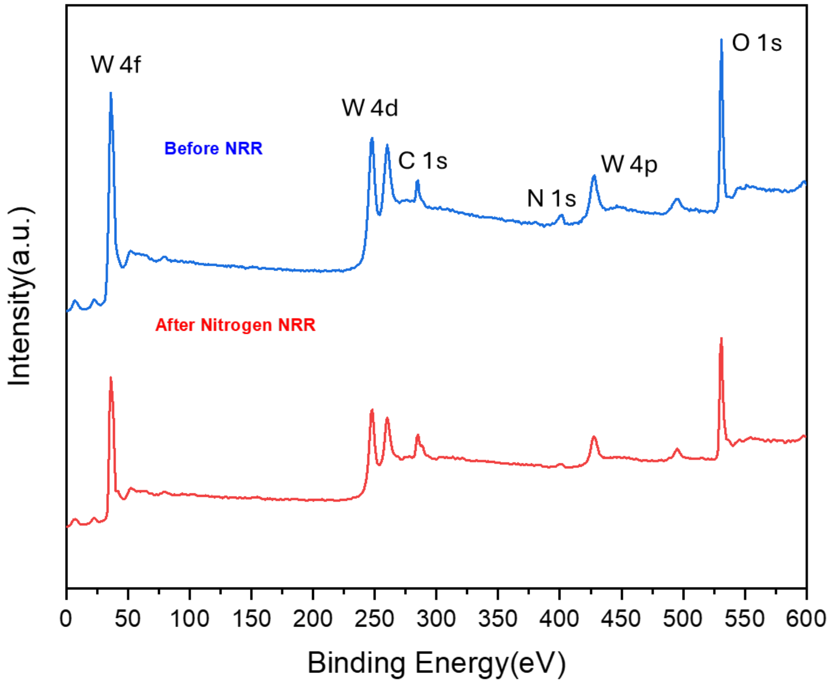

Figure S7. XPS spectra of WO_3/WO_xN_y before and after NRR.

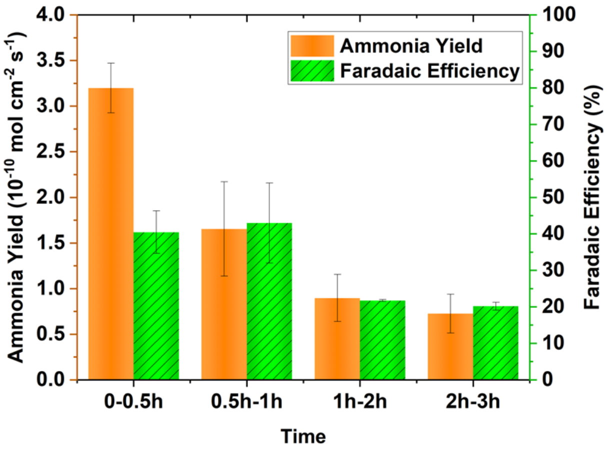

Figure S8. Specific yield rate and faradaic efficiency of WO_3-H_2/N_2-2h at -0.15V in three hours cycle.

Reference:

(1) Gao, L.; Wang, X.; Xie, Z.; Song, W.; Wang, L.; Wu, X.; Qu, F.; Chen, D.; Shen, G. High-performance energy-storage devices based on WO_3 nanowire arrays/carbon cloth integrated electrodes. *Journal of Materials Chemistry A* **2013**, *1* (24), 7167-7173. DOI: 10.1039/C3TA10831G
